# Online closed‐loop real‐time tES‐fMRI for brain modulation: A technical report

**DOI:** 10.1002/brb3.2667

**Published:** 2022-09-22

**Authors:** Beni Mulyana, Aki Tsuchiyagaito, Masaya Misaki, Rayus Kuplicki, Jared Smith, Ghazaleh Soleimani, Ashkan Rashedi, Duke Shereen, Til Ole Bergman, Samuel Cheng, Martin P. Paulus, Jerzy Bodurka, Hamed Ekhtiari

**Affiliations:** ^1^ Laureate Institute for Brain Research Tulsa Oklahoma USA; ^2^ Electrical and Computer Engineering University of Oklahoma Tulsa Oklahoma USA; ^3^ Department of Biomedical Engineering Amirkabir University of Technology, Tehran, Iran; ^4^ Iranian National Center for Addiction Studies Tehran University of Medical Sciences Tehran Iran; ^5^ The Graduate Center of the City University of New York New York New York USA; ^6^ Neuroimaging Center (NIC) University Medical Center of the Johannes Gutenberg University Mainz Germany; ^7^ Leibniz Institute for Resilience Research (LIR) Mainz Germany; ^8^ Stephenson School of Biomedical Engineering University of Oklahoma Norman Oklahoma USA; ^9^ Department of Psychiatry and Behavioral Sciences University of Minnesota Minneapolis Minnesota USA

**Keywords:** closed‐loop, executive control network, fMRI, frontoparietal network, optimization, precision medicine, tES

## Abstract

Recent studies suggest that transcranial electrical stimulation (tES) can be performed during functional magnetic resonance imaging (fMRI). The novel approach of using concurrent tES‐fMRI to modulate and measure targeted brain activity/connectivity may provide unique insights into the causal interactions between the brain neural responses and psychiatric/neurologic signs and symptoms, and importantly, guide the development of new treatments. However, tES stimulation parameters to optimally influence the underlying brain activity may vary with respect to phase difference, frequency, intensity, and electrode's montage among individuals. Here, we propose a protocol for closed‐loop tES‐fMRI to optimize the frequency and phase difference of alternating current stimulation (tACS) for two nodes (frontal and parietal regions) in individual participants. We carefully considered the challenges in an online optimization of tES parameters with concurrent fMRI, specifically in its safety, artifact in fMRI image quality, online evaluation of the tES effect, and parameter optimization method, and we designed the protocol to run an effective study to enhance frontoparietal connectivity and working memory performance with the optimized tACS using closed‐loop tES‐fMRI. We provide technical details of the protocol, including electrode types, electrolytes, electrode montages, concurrent tES‐fMRI hardware, online fMRI processing pipelines, and the optimization algorithm. We confirmed the implementation of this protocol worked successfully with a pilot experiment.

## Graphical abstract

Here, we assessed tolerability/safety/noise concerns and methodological aspects of a real‐time closed‐loop tACS‐fMRI trial. We discussed the risk of electrode heating and localization of the electrodes, while people are lying down inside the scanner, MRI conditional stimulator setup, RF filters, and wire outing patterns, the amount/type of contact medium, the timing of concurrent tACS within the fMRI paradigm, optimization algorithm and parameters search space to optimally target the frontoparietal network. The initial results of feasibility and applicability of closed‐loop tACS‐fMRI are reported, and potential hypotheses for the outcomes are summarized.

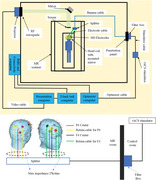

## 1 INTRODUCTION

Transcranial electrical stimulation (tES) provides electric current stimulation over the scalp to modulate specific brain regions’ neural activity or their functional connectivity (Bikson et al., 2019). This method can be concurrently combined with functional magnetic resonance imaging (fMRI). Such tES‐fMRI combination has several technical advantages (Saiote et al., 2013; Williams et al., 2017) compared with (1) sequential fMRI‐tES‐fMRI in terms of the ability to investigate ongoing brain activity and (2) simultaneous tES‐electroencephalography (EEG) in terms of higher spatial resolution and fewer problems with stimulation artifacts. A major advantage of concurrent tES with fMRI is that we can stimulate several regions of the brain by tES (i.e., two nodes of a network with conventional or high definition (HD) electrode montages) and evaluate its online stimulation effect by fMRI to reveal associations between brain stimulation and whole‐brain activity/connectivity (Bächinger et al., 2017; Cabral‐Calderin et al., 2016; Violante et al., 2017; Vosskuhl et al., 2016).

tES is a noninvasive brain stimulation (NIBS) technique including direct (tDCS), alternating current (tACS), and random noise stimulation (tRNS) (Bikson et al., 2019). Although all tES methods can target large‐scale brain networks, tACS has the unique potential to modulate oscillations within or between the large‐scale brain networks using alternating currents at a chosen frequency and phase difference between network nodes to interact with synchronization‐based functional connectivity (Ruffini et al., 2014). The blood oxygenation‐level‐dependent (BOLD) fMRI signal relies on the blood flow response to brain neuronal activity, which is much slower than the electrophysiological activity of the individual neuron. The BOLD response starts to increase a few seconds after the respective change in neural activation. However, the presence of correlated BOLD signal fluctuations at rest‐state fMRI (e.g., resting‐state networks) across brain areas may result from oscillatory synchronization facilitating communication between those regions (Buzsáki & Draguhn, 2004; Canolty & Knight, 2010), as supported by findings from concurrent EEG‐fMRI studies demonstrating the association of electrophysiological neuronal oscillation as measured by EEG and BOLD signal with large spatial scale synchronization (Mantini et al., 2007; Whitman et al., 2013; Yuan et al., 2012, 2016). Therefore, fMRI functional connectivity across various brain regions may serve as a proxy‐marker to measure internal co‐oscillatory electrophysiological synchronization of those regions. External oscillatory stimulation (e.g., at a frequency range matching EEG rhythmic activity: 0.1–40 Hz or even higher) above several cortical regions using multisite tACS has been demonstrated to increase internal oscillatory synchronization and functional connectivity between brain regions (Cabral‐Calderin et al., 2016; Kuo & Nitsche, 2012; Moisa et al., 2016; Violante et al., 2017; Weinrich et al., 2017a; Williams et al., 2017; Zoefel et al., 2018).

However, determining the ideal configuration of a multisite tACS system aimed at modulating brain networks is complex, as the effects of tACS are highly dependent on the stimulation parameters such as electric current stimulation intensity, frequency, inter‐regional phase differences, selection of electrode locations, and individual differences in brain structure (Antal & Paulus, 2013). For example, a plausible range of stimulation frequencies (usually 1–150 Hz, up to 5 kHz based on the literature) (Kunz et al., 2017) and phase differences (0–359°) between stimulation sites (Lorenz et al., 2019) results in a wide range of possibilities. Establishing optimization algorithms would aid the clinical application of tACS. More precisely, an online fMRI measurement will enable us to establish an empirically optimized algorithm by identifying the stimulation parameters (i.e., frequency and phase differences in this study) which maximize the targeted brain network activity/connectivity (i.e., temporal correlations between BOLD signal changes in two target regions). Moreover, applying multielectrode configurations or HD montages (Datta et al., 2009) has been shown to result in more focal electric field distribution patterns (Alam et al., 2016; Villamar et al., 2013), which will also allow unique combinations of electrode locations combined with optimized stimulation parameters to more focally target specific cortical regions (Dmochowski et al., 2011).

Here, we report our recently developed protocol of closed‐loop tACS‐fMRI with optimizing the stimulation frequencies and phase differences of tACS for two brain regions, frontal and parietal areas, to maximize the frontoparietal synchronization (FPS) (Saturnino et al., 2017) in individual participants. We developed, for the first time, a fully closed‐loop tACS‐fMRI using an MRI‐compatible high‐definition tACS (HD‐tACS) setup with two sites 4 × 1 ring montages, real‐time fMRI evaluation of FPS, and online optimization of tACS parameters. We first present the details of the tACS‐fMRI equipment, including electrode types, electrolytes, electrode montages, and concurrent tES‐fMRI hardware. We tested the safety and quality of our closed‐loop tACS‐fMRI setting regarding temperature under electrodes, patient comfort, sensation, and artifact in fMRI signal. Then, we present the detailed protocol of the online closed‐loop tACS optimization with real‐time fMRI and a numerical optimization method. Lastly, we confirmed the feasibility of our protocol implementation for pilot participants. Expected outcomes and hypotheses for future studies are also discussed.

## 2 CLOSED‐LOOP tACS‐fMRI SETUP

### 2.1 System overview

**FIGURE 1:**
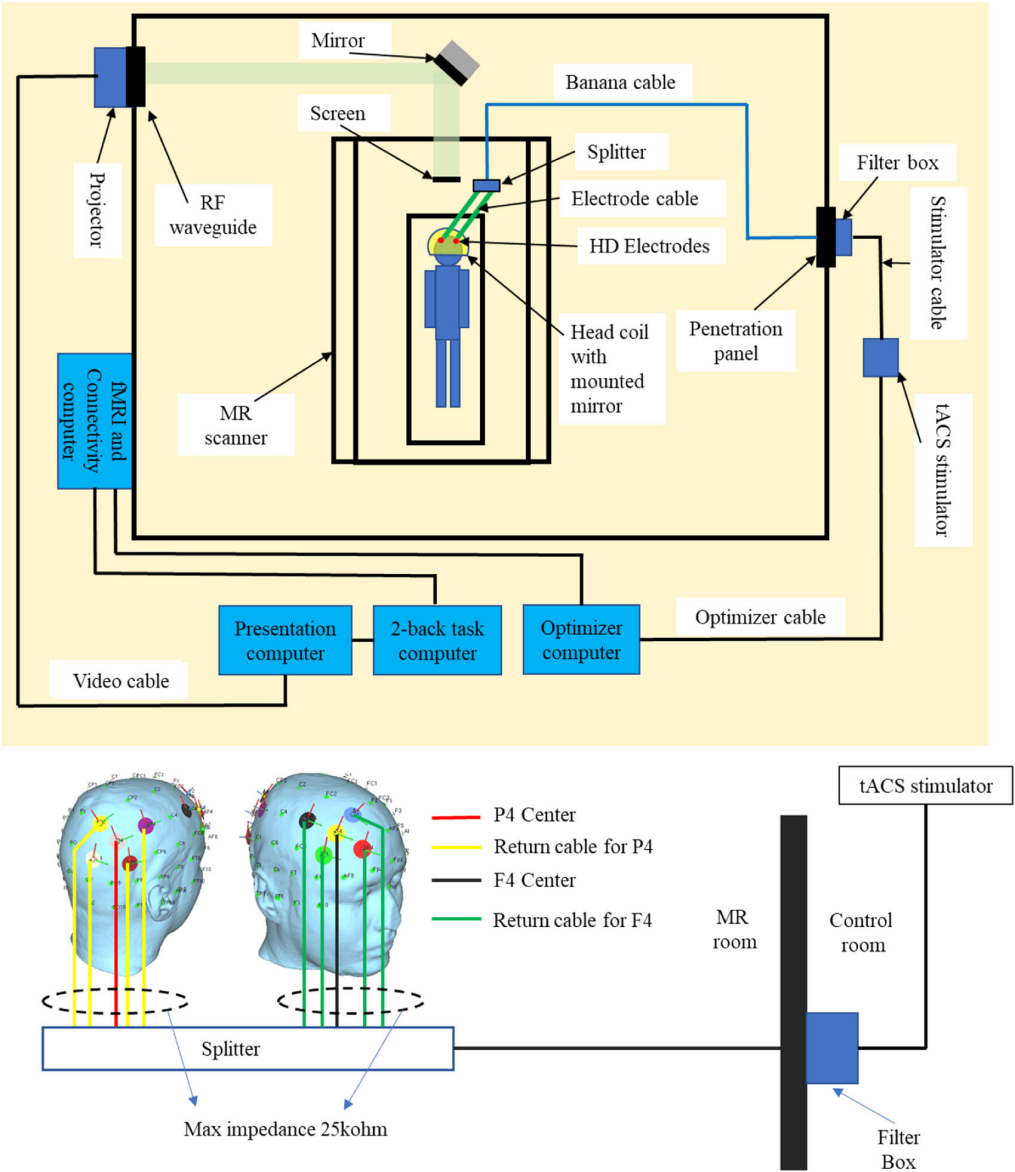
Closed‐loop tES‐fMRI setup. The participant is capped with 10 high‐definition (HD) electrodes in a frontoparietal montage, then lays down inside the MRI room to get tACS stimulation concurrent with fMRI scanning. During fMRI scanning, fMRI connectivity computer sends frontoparietal connectivity to the 2‐back task computer and the optimizer computer. 2‐back task computer is connected to the presentation computer to display 2‐back task on the screen inside the MRI room for the participant. The optimizer calculates the optimal tACS parameters for improving participant frontoparietal functional connectivity. Then, the optimizer sends the tACS parameters through the optimizer cable to the tACS device. The tACS device is connected to the filter box that is attached on the penetration panel using a stimulator cable. Then, the filter box is connected through a banana cable to the participant's frontoparietal sites via 10 HD electrodes to give the stimulation.

Figure 1 shows an overview of the closed‐loop tACS with a concurrent fMRI system. The tACS stimulation was applied using a battery‐driven MRI‐compatible Starstim AC‐Stimulator (https://www.neuroelectrics.com/products/starstim/starstim‐r32/). The tACS device is positioned outside the magnetic field in the operator room (Figure 1). The stimulation current is channeled into the scanner bore via a filter box (MECMRI‐Series, 2018) attached to the penetration panel that filters out radio frequency (RF) noise (7−1000 MHz) and high magnetic fields from the scanner.

### 2.2 tACS electrodes

The Starstim R32 tACS device uses rubber electrodes embedded in sponge pockets with saline solution as a conductive material between the electrode and scalp. Although this electrode solution is more comfortable for participants compared to conductive gel, using saline solution has many disadvantages, such as: (1) saline solution evaporates quickly, which makes it difficult to maintain safe and low impedance during long duration experiments; (2) saline solution easily spreads out and has a greater risk of short‐circuit between electrodes, which will not provide accurate stimulation over the desired sites of cortical area; and (3) the sponge is made of textile sponge and the contact with the carbon rubber could be loose.

**FIGURE 2:**
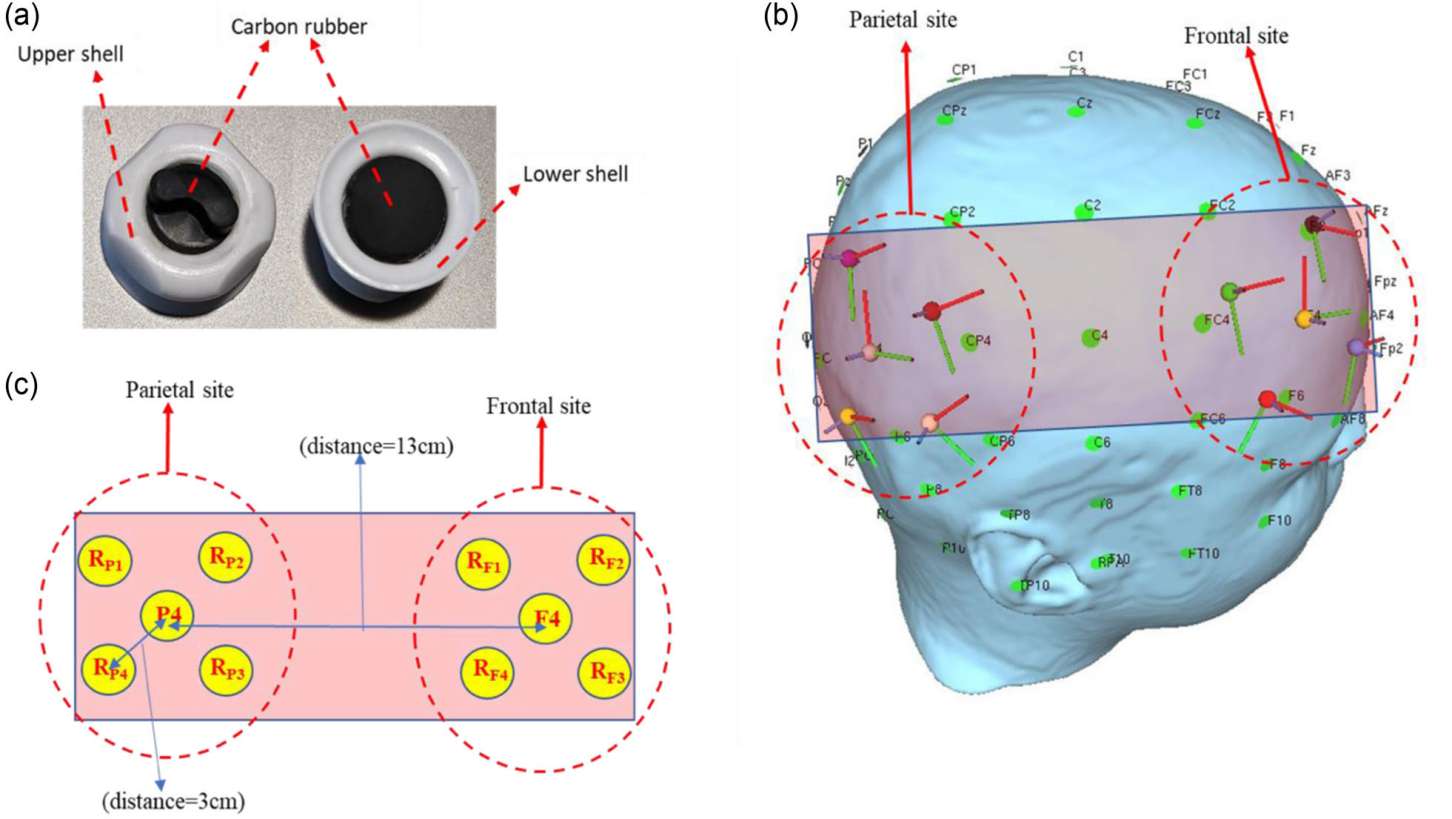
MR compatible HD electrodes and montage settings for this study. (a) MR compatible high definition (HD) electrodes; (b) head model of equidistance center‐return (3 cm) electrode placement; (c) plane surface of equidistance (3 cm) electrode placement, with a distance of 13 cm between sites. To simplify analyses, we ignored the head curvature, and drew the plane surface of the head where the electrodes are positioned, as shown in (b and c) above. The electrodes side view is shown in Figure S1.

To overcome these potential disadvantages of saline solution and also to take advantage of the focality of HD electrodes, we created MRI compatible rubber HD electrodes (circular pad with radius 10 mm and 1 mm thickness, electrode material: carbon rubber and plastic shell) (Figure 2a). We removed the carbon rubber cores from MRI Sponstim (model: NE026MRI, brand: Neuroelectrics) and placed them inside next‐generation (NG) Pistim's shells (model: NE029, brand: Neuroelectrics) to replace the metal part (Ag/AgCl) of the shells. We applied highly conductive gel/paste (Piervirgili et al., 2014) (model: Abralyt HiCl, brand: Easycap) between those MRI‐compatible electrodes and the scalp to improve contact conductivity. Our electrode shell construction has a dome structure so that it avoids gel spreading out over the scalp and will be a better setting compared with electrodes embedded in sponge pockets soaked with saline solution. The electrode is made from a nonmagnetic material (carbon rubber), and it connects to an MRI electrode cable that is also made from a carbon rubber (model: NE046c, brand: Neuroelectrics) with distributed low‐conductivity to reduce stray fields in magnetic resonance current density imaging (MRCDI) (Gregersen et al., 2021). This setting will allow us to minimize the possibility of spurious electric field. We used textile caps with holes indicating places for electrode positioning (model: Neoprene Headcap/NE019, brand: Neuroelectrics).

### 2.3 Electrode montage

We targeted the frontoparietal network to maximize the FPS with tACS stimulation. This network is within the executive control network (ECN) and is involved in sustained attention, complex problem‐solving, and working memory (Menon, 2011). Specifically, the present protocol targeted the right middle frontal gyrus and right inferior parietal cortex as important nodes of the frontoparietal network, which are approximated by electrode positions at F4 [49.65, 53.71, 72.15] (mm in MNI space) and P4 [48.73, −84.52, 66.10] of the 10‐10 EEG system using Ernie model in the SimNIBS software (Saturnino, Madsen, & Thielscher, 2019).

The current of the center electrodes was fixed to 1 mA‐peak value. Although higher stimulation intensities (up to 4 mA) could result in a higher neural response (dose–response relationship) (Karabanov et al., 2019; Kessler et al., 2012; O'Connell et al., 2012), we used a lower current intensity (1 mA) in this first closed‐loop tACS fMRI pilot study. Previous tES fMRI studies used similar current (i.e., 1 mA) or even lower (Antal et al., 2008; Moliadze et al., 2012; Splittgerber et al., 2020; Violante et al., 2017). Higher doses could be evaluated after confirming the subjects’ tolerance.

**FIGURE 3:**
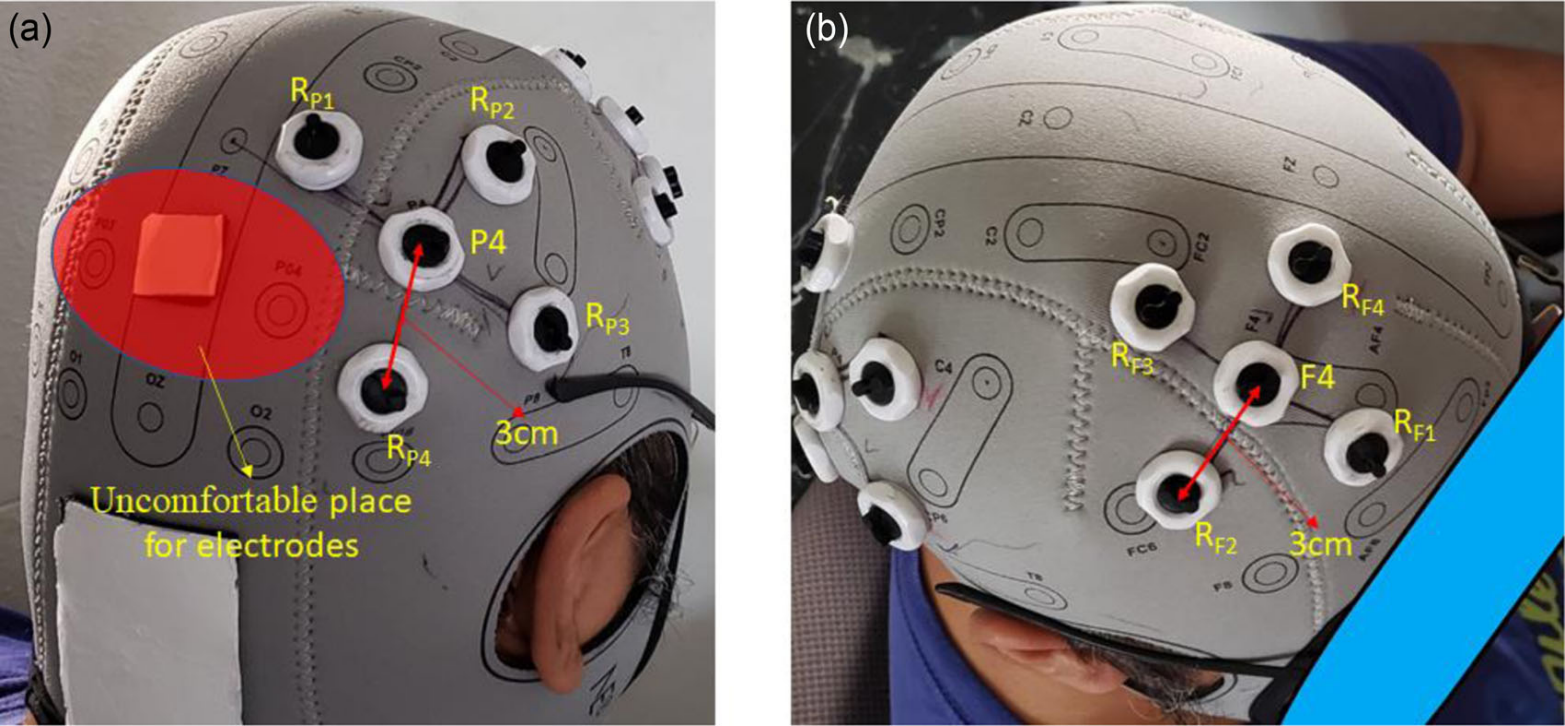
Overview of the frontoparietal montage with 10 HD electrodes. (a) Montage of five high‐definition (HD) electrodes on the parietal site with equidistant (3 cm) center and return electrodes. The red highlighted area is a subject uncomfortable area where we need to avoid placing the electrodes due to the constant head pressure when subjects lie down on the MRI table; (b) Montage of five HD electrodes on the frontal site with equidistant (3 cm) center and return electrodes. Red area indicates the area needs to be aware of for MRI scanning due to the pressure when subjects lie down on the MRI table.

The current of each return electrode (four return electrodes on each side) was set to 0.25 mA. Return‐electrode placement for F4 and P4 sites was designed to be at an equal center‐return distance (3 cm) in order to reduce gel bridging (short‐circuit) and to reduce the electrical shunt effect in the anti‐phase condition, explained in section 2.5. Return‐electrode coordinates for the F4 site are: R_F1_ = [51.35, 28.51, 86.09], R_F2_ = [25.05, 58.87, 87.63], R_F3_ = [42.95, 74.26, 51.96], and R_F4_ = [64.83, 41.57, 52.64] (Figure 3b) and return‐electrode coordinates for P4 site are: (R_P1_ = [49.02, −95.93, 38.49], R_P2_ = [25.63, −89.69, 84.28], R_P3_ = [52.57, −62.68, 85.96], and R_P4_ = [65.54, −67.52, 51.97] (Figure 3a) in the MNI coordinates aligned from the Ernie model from SimNIBS software. Care was taken to avoid placing the return electrodes around PO4, as this electrode site would result in uncomfortable pressure on the back of the subject's head when lying on the MRI table (the red area in Figure 3a).

### 2.4 tACS capping

**FIGURE 4:**
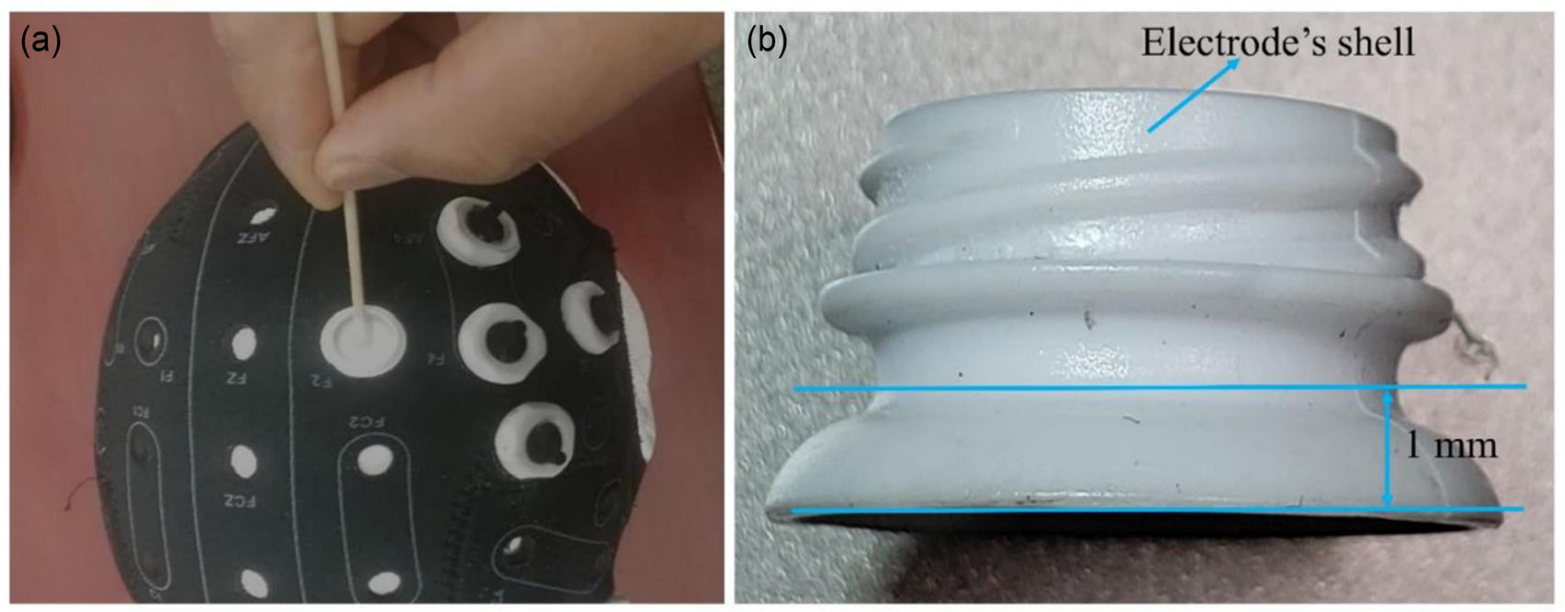
Peripheral cautions for dual site HD montage capping. (a) Swab evenly and gently using a cotton swab with isopropyl alcohol on the scalp area inside the electrode shell. Repeat three or four times. (b) After 10 high definition (HD) electrodes’ shells are attached into the holes on the cap referring to the montage location, the gel is spread evenly across the scalp inside the shell. The layer of gel should not exceed 1‐mm thickness or 0.5‐ml gel volume to reduce excessive leakage of gel, which could make a short‐circuit between nearby electrodes.

Before applying gel, we checked that there were no tattoos, scars, or active skin irritation around the electrode location. Afterward, we cleaned the scalp area in the electrode shell with isopropyl alcohol (IPA) using a cotton swab. This is to clean the scalp area where the electrodes will be installed, so that dust and oil in the area will be removed to make a low impedance contact between the electrode and the scalp. We dipped the cotton swab into the IPA, then swabbed the scalp under the electrode shell with the cotton swab evenly and gently (Figure 4a). We repeated these procedures three or four times. Then we applied Abralyt HiCl gel to the scalp area inside the electrode's shell so that the amount of gel avoids a short‐circuit between the electrodes. If a short‐circuit occurs between electrodes, tACS will not work as intended. The gel must be spread evenly across the scalp inside the shell, and the level of gel should not exceed the thickness shown in Figure 4b, which is about 1 mm or the amount of 0.5 ml.

### 2.5 Electric field of the montage

Electric field derivation for in‐phase and antiphase conditions in the frontoparietal montage can be found in Supporting Information A. Derivations show that the electric field on the in‐phase condition from our montage will appear under the frontal and parietal electrodes but not between them. Any appearances of the electric field between the sites are the electric shunt effect (Saturnino, Madsen, et al., 2017). Electric shunt increases the stimulated area and decreases focality. This is not desirable if we need to focus stimulation over a specific region (e.g., frontal and parietal areas in the frontoparietal network). Therefore, the in‐phase condition is relatively safe from shunt condition. Our montage with 13‐cm distance between each site does not show the electric shunt effect between their sites (Figure 4a,b). During the antiphase condition, there is a possibility of the electric shunt effect between each site (see Equation (6) in Supporting Information A). Therefore, based on Equation (6), and to avoid the shunt effect, we need to pay attention to (i) ensure sufficient distance between the return electrodes of the two sites and (ii) position the return electrodes as close as possible to their center electrode (*d*_1_ ≈ *d*_2_ ≈ *d*_3_) but not too close to prevent too much shunting effect via the skin between the center and surround electrodes (Neri et al., 2020).

In the montage, it is feasible to establish a return‐to‐center distance of 3 cm, resulting in a gap of 1 cm between the edges of the center and return electrodes (each of them having a diameter of 2 cm). With this montage, the return‐to‐center electrode distance is relatively small, and the return‐to‐return electrode distance between frontal and parietal sites is relatively large so that the shunt effect between frontal and parietal sites in the antiphase condition is minimized. From our montage, by using Equation (6) (in Supporting Information A) and data; 2d_2_ = distance from F4 to P4 around 13 cm (https://www.biosemi.com/headcap.htm), 2d_2 _= d_1_+ d_3_ = 13 cm and gray matter conductivity = 0.275 S/m (Wagner et al., 2004), the maximum electric field in the gray matter midway between the two sites was 0.04 V/m in the antiphase condition, which is below than 0.1 V/m. The electric field intensity as 0.1 V/m is considered as the threshold for measurable physiological effects in neurons (Huang et al., 2017; Jefferys et al., 2003; Ozen et al., 2010), and with this montage, the electric field between the sites can be negligible even in the antiphase condition.

**FIGURE 5:**
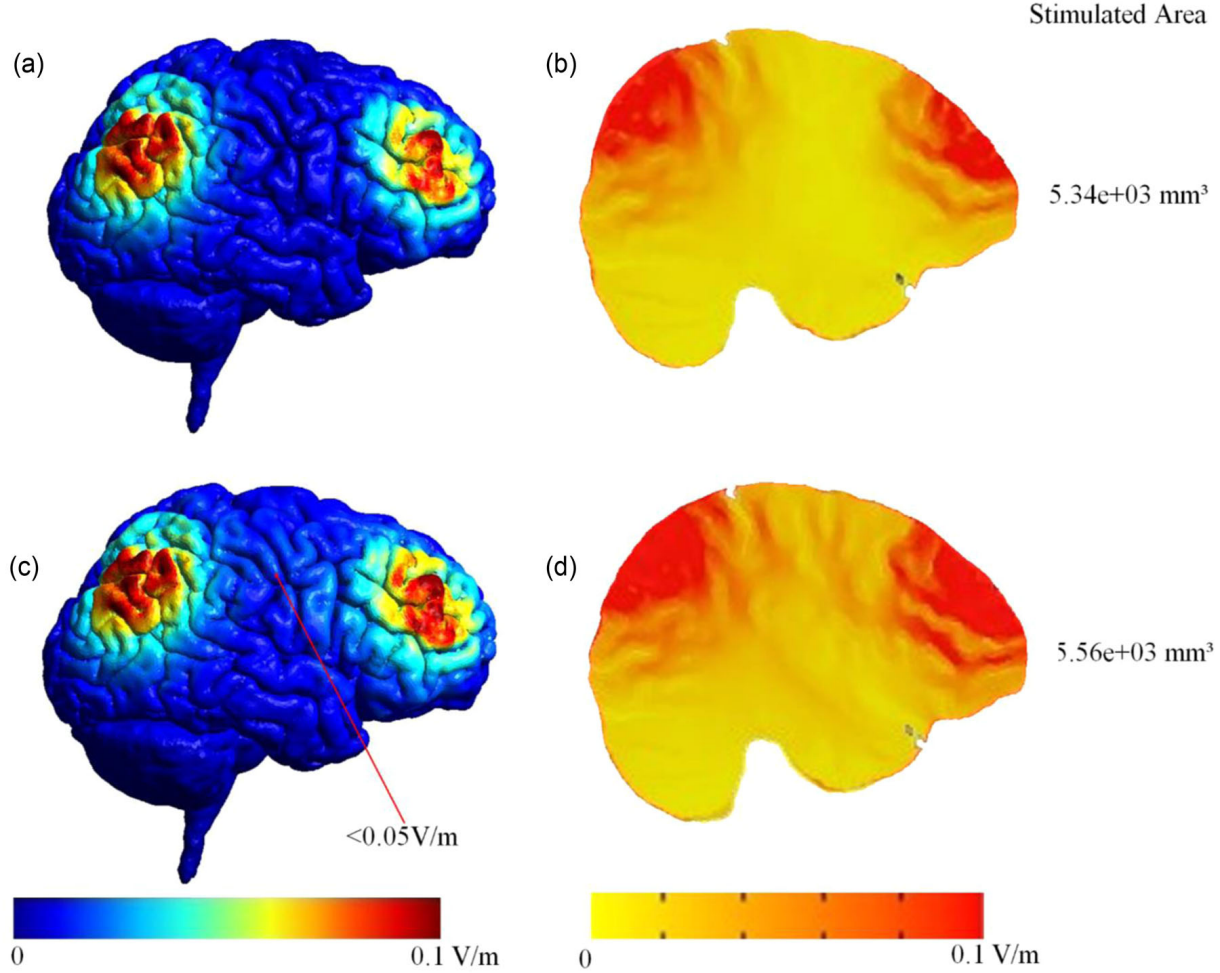
Surface‐ and volume‐based comparison of in‐ and antiphase conditions. The cortical surface (surface‐based electric field distribution) is calculated by SimNIBS (a and c) and could be interpolated into a NIfTI volume and transformed to MNI space (volume‐based electric field distribution). Therefore, we can analyze the electric field in each voxel using AFNI software (b and d). (a) and (b) Surface‐ and volume‐based simulation result of the in‐phase condition; (c) and (d) Surface‐ and volume‐based simulation result of the antiphase condition. The stimulated area in the antiphase is 4.12% larger than in the in‐phase condition due to the electric shunt effect. In the antiphase condition, the electric shunt effect can be seen as a stronger electric field (red color) between sites (d).

The electric field in the cortical target regions of interest in the frontal and parietal cortex was provided by SimNIBS. The top percentiles of the electric field intensity in 99.9% was 9.22e‐02 V/m or close to 0.1 V/m, which appeared on the cortical surface under the center electrode for each site (frontal and parietal) (Figure 5). Our simulation results indicate that the electric field obtained on the cortical areas under each site was high to capture measurable physiological effects in neurons, and the shunting effect between the center and their surrounding return electrodes can be negligible. To test these hypotheses in more detail in silico, we simulated the electric field in the brain using SimNIBS 3.2 software (Saturnino, Puonti, et al., 2019; Thielscher et al., 2015). SimNIBS 3.2 uses the finite element mesh (FEM) method to calculate the electric field on every tetrahedron element mesh in every brain segmentation. It can be interpolated onto the cortical surface (surface‐based electric field distribution) or interpolated into a NIfTI volume and transformed to MNI space (volume‐based electric field distribution). Therefore, we can analyze the electric field in each voxel. SimNIBS also provides information about the focality of the stimulated area, which is defined as the gray matter volume with an electric field greater or equal to 75% of the peak value. To avoid the effect of outliers, we defined the peak value as the 99.9th percentile. The smaller the value of this volume metric, the more focal the electric field in the brain.

Figure 5a,b depict the intensity of the electric field on the cortical surface, on volumetric sagittal view, and the stimulated area for F4‐P4 in‐phase. Meanwhile, Figure 5c,d show the intensity of the electric field on the cortical surface, on volumetric sagittal view, and the stimulated area for F4‐P4 antiphase. Figure 5a–d indicate that the electric field was focused under frontal and parietal sites as predicted by Equation (4). However, in the antiphase condition, the electric field between sites appears stronger than in the in‐phase condition, and also the stimulated area was larger than the in‐phase condition (in‐phase stimulated area = 5.34 × 10^3^ mm^3^, antiphase stimulated area = 5.56 × 10^3^ mm^3^, percent change at antiphase from in‐phase = 4.12%). This is caused by the electric shunt effect. As predicted by Equation (6), the maximum shunt effect on the cortical surface was less than 0.05 V/m (Figure 5c). The electric shunt effect for the antiphase condition was not overly large (i.e., the stimulated area only increases by 4.12% compared to the in‐phase condition), so the shunt effect can be neglected.

## 3 tACS‐fMRI QUALITY CHECK AND SAFETY

### 3.1 Background

Before applying tACS‐fMRI, it is necessary to verify the impact of tACS‐fMRI on fMRI image quality and safety. Reliable and safe setups for the application of simultaneous tACS‐fMRI are well known (Chaieb et al., 2014; Frank et al., 2010; Gbadeyan et al., 2016; Loo et al., 2011; Poreisz et al., 2007; Williams et al., 2017). However, there is no published evidence on the safety of simultaneous tACS‐fMRI with dual‐site HD montages. Therefore, we first scanned a watermelon (in Supporting Information A) to test MRI artifacts and noise, and then conducted a human scan to measure the temperature changes during tACS‐fMRI in order to prove that combined tACS‐fMRI has no aversive impact on human safety and image quality. We aimed to: (1) examine whether tACS stimulation significantly induces any artifacts or increases noise on MRI/fMRI images, and (2) to conduct a tACS safety test regarding the scalp temperature under the stimulation electrodes during concurrent tACS stimulation during fMRI. Details of MRI artifacts, fMRI noise testing methods, and a temperature test can be found in Supporting Information B.

### 3.2 MRI/fMRI safety testing results

#### 3.2.1 MRI artifacts and fMRI noise

**FIGURE 6:**
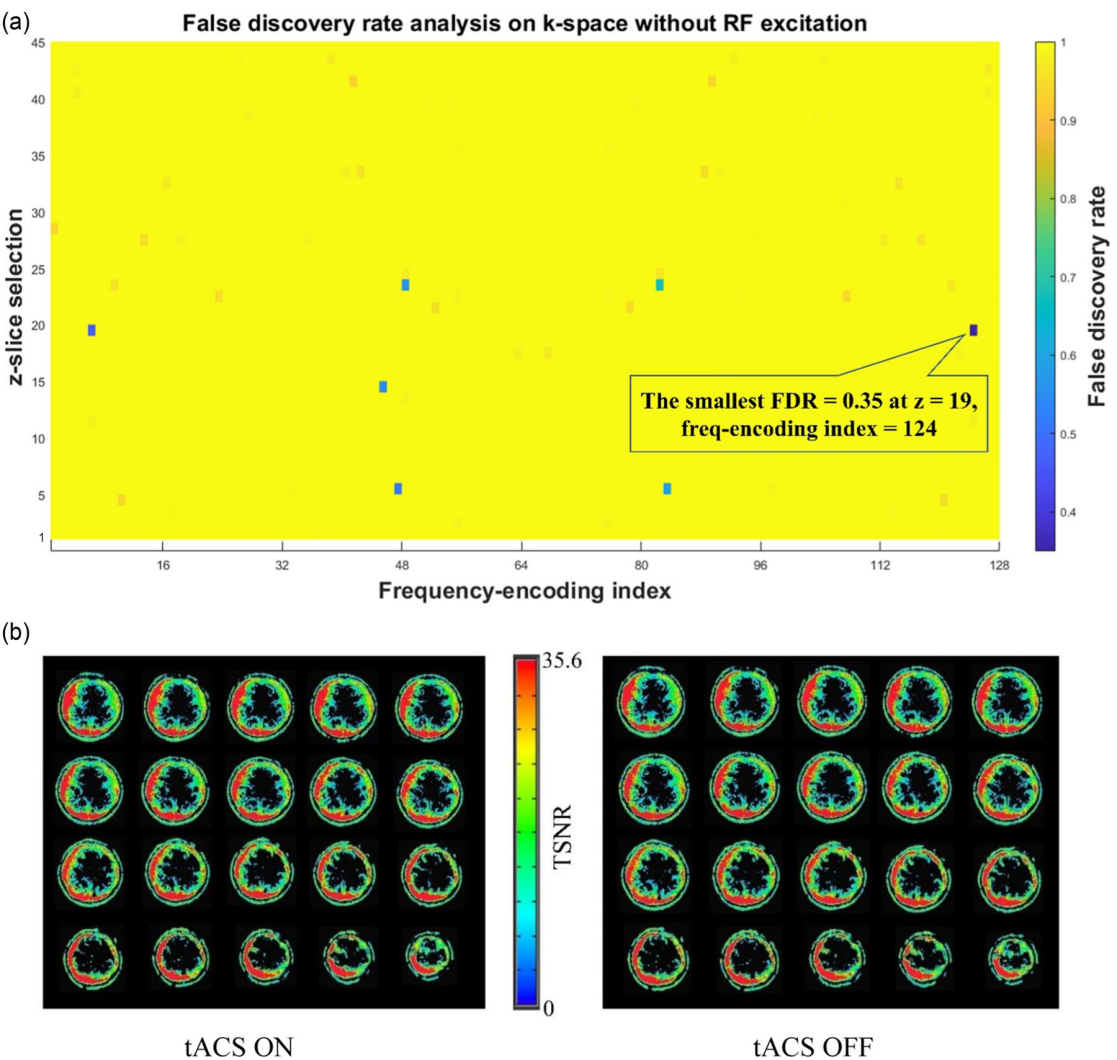
Results of the noise test influencing fMRI signal (EPIs). (A) FDR‐corrected *p*‐value in the k‐space without‐RF excitation. The smallest FDR‐corrected voxel‐wise *p* value = .35 which is larger than .05. It means tACS stimulation did not create significant artifacts. (b) Voxel‐wise temporal signal‐to‐noise ratio (TSNR) from stimulation ON and OFF. A visual inspection corroborates here indicated no tACS‐related artifact observed in the EPI images. It is also confirmed by the voxel‐wise analysis (with‐RF) where the smallest FDR‐corrected voxel‐wise *p* value = .312 > .05.

We scanned a watermelon with concurrent tACS‐fMRI to evaluate the tACS noise that is free from the effect of a neural activation signal. Detailed procedures of this artifact and noise test can be found in Supporting Information B. We first obtained k‐space data without RF excitation pulse. Since no echo signal was emitted without RF excitation, we could measure only the tACS noise received by seeing the k‐space data. We collapsed the phase encoding direction by averaging, and made a frequency‐by‐slice k‐space image for each volume. Figure 6a shows the FDR‐corrected *p*‐values for the t‐test for the received signals between the stimulation ON and OFF period. The smallest FDR‐corrected *p*‐value was .35, indicating that tACS did not produce significant noise in the received signal.

Second, we scanned a watermelon in the same way but with a RF excitation pulses. We performed GLM analysis to test the signal difference between the stimulation ON and OFF period in the signal time‐course. We found no voxel had a significant difference between the stimulation ON and OFF period (the smallest FDR corrected *p*‐value was .312). We also compared mean value and SD of time series in each voxel within the ROIs (F4 and P4), and found no significant difference between tACS ON and OFF (ON: mean = 1210.70 and OFF: mean = 1210.50, *t*[223] = 0.4, *p *= .72; ON: SD = 2.24 and OFF: SD = 2.55, *F*[150,75] = 1.14, *p *= .27; Figure 6b). The temporal signal‐to‐noise ratio (TSNR) map is shown in Figure 6. These results indicated that tACS did not produce significant noise in the echo signal and the fMRI image time‐series.

#### 3.2.2 Temperature measurement results

**FIGURE 7:**
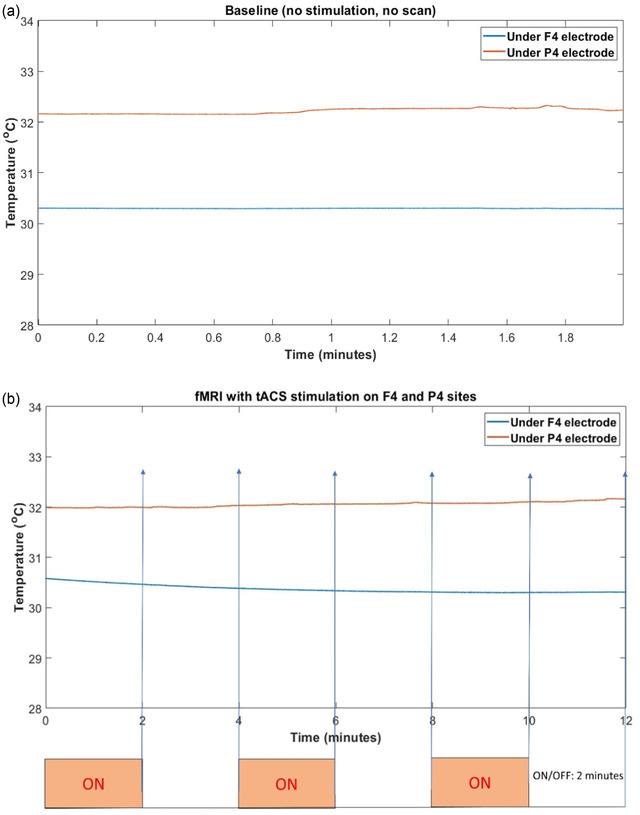
Safety test using temperature records under the electrodes. (a) Baseline temperature on the scalp at F4–P4 electrodes when there is no tACS scan. The baseline scalp temperatures prior to scanning and tACS stimulation are stable below 33°C; (b) Temperature during fMRI with and without tACS under F4–P4, 2 min ON/2 min OFF for 12 min. The temperatures did not significantly change regardless of the tACS stimulations ON or OFF. Furthermore, the scalp temperatures under the electrodes are below the upper limit human body temperature (37.5^o^C) during a 12 min fMRI (EPI) scan. There is no issue with patient safety in terms of temperature changes during tACS‐fMRI.

Next, we scanned a human subject to examine the temperature change due to tACS during fMRI. Details of the test procedure can be found in Supporting Information B. The normal human body temperature is typically observed in a range from 36.5°C to 37.5°C (Hutchison et al., 2008; Mackowiak et al., 1992). The baseline scalp temperatures prior to scanning and tACS stimulation were stable below 33°C (F4: mean = 30.30, SD = 0.003; P4: mean = 32.22, SD = 0.05) (Figure 7a). The EPI scan did not cause a substantial heating effect at the tACS electrodes (F4: mean = 30.37, SD = 0.08; P4: mean = 32.05, SD = 0.05) (Figure 7b). Moreover, the temperatures did not significantly change with the tACS stimulation (F4 ON; mean = 30.39, SD = 0.11: F4 OFF; mean = 30.35, SD = 0.05; z = 0.68, *p* = .49: P4 ON; mean = 32.04, SD = 0.04: P4 OFF; mean = 32.07, SD = 0.05; z = −0.59, *p* = .56). Furthermore, the scalp temperatures under the electrodes are below 37.5°C during a 12‐min EPI scan, confirming that there is no issue with patient safety in terms of temperature change during concurrent tACS‐fMRI in the current experimental set‐up.

## 4 ONLINE OPTIMIZATION OF FRONTOPARIETAL SYNCHRONIZATION WITH CLOSED‐LOOP tACS‐fMRI

Frontoparietal connectivity within the executive control network is considered one of the main therapeutic targets for network‐based brain stimulation in different psychiatric disorders, for example, depression and substance use disorders (Ekhtiari et al., 2016; Fischer et al., 2016). Here, we provide a detailed protocol for an online FPS closed‐loop tACS‐fMRI study. The protocol is specifically designed to determine the optimal frequency and phase difference of dual‐site tACS to enhance frontoparietal connectivity during stimulation. Closed‐loop tACS‐fMRI is composed of three processes: tACS stimulation with concurrent fMRI, online evaluation of the tACS effect on FPS with real‐time fMRI, and optimization of the stimulation parameters according to the online FPS evaluation. A loop of these processes is expected to find the optimal tACS stimulation parameters for maximizing FPS. The cognitive task applied to a participant during the loop is also critical to reliably evaluate the tACS effect and successful optimization. The details of each process are described below.

### 4.1 Overview of the session schedule

**FIGURE 8:**
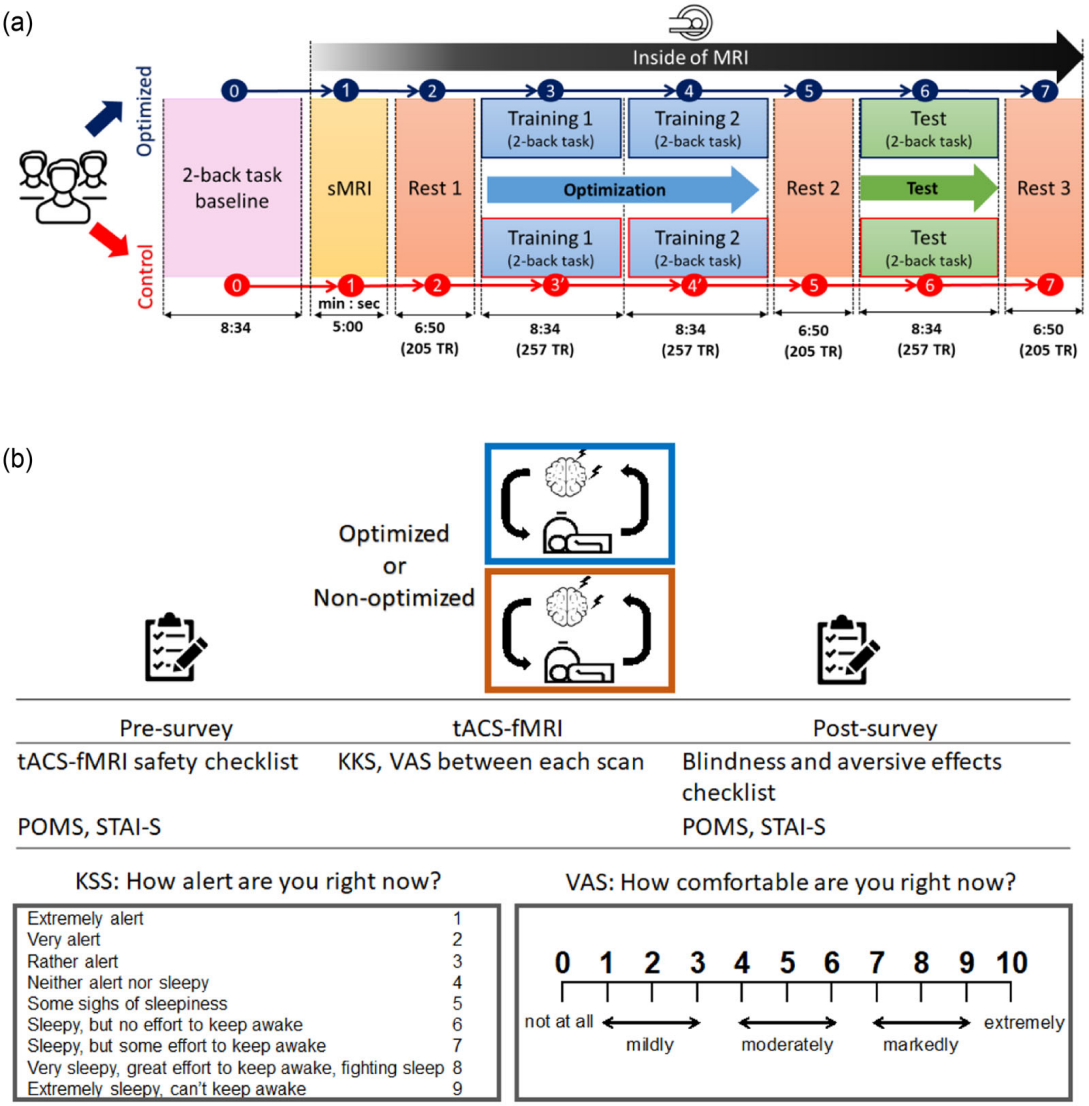
Study design. (a) A procedure of the online frontoparietal synchronization optimization protocol with a closed‐loop tACS‐fMRI. TR = time repetition, 2 s. (b) An overview of the session. *Abbreviations*: KSS, Karolinska Sleepiness Scale; POMS, Profile of Mood States Scale; STAI‐S, State‐trait Anxiety Inventory‐State version; VAS, Visual Analogue Scale asking one's comfortableness.

Figure 8 shows procedures of online FPS optimization protocol. The stimulation sessions are divided into two runs (training and testing). The training runs determine the optimal frequency and phase difference parameters that produce the highest frontoparietal network connectivity while subjects perform a cognitive task (i.e., a 2‐back task) (Figure 8a). An anatomical scan (sMRI) is applied before the FPS optimization session as a reference image for the regions of interest of functional connectivity calculation in real‐time fMRI. Resting‐state scans (rsfMRI) are also applied before and after the FPS optimization sessions and after the test session. Participants are asked to answer several self‐surveys before and after the FPS sessions to evaluate the feasibility and the side‐effects of the FPS session (Figure 8b). Further details of the study protocol are described later in the Study design section.

### 4.2 Cognitive task during the FPS optimization

Prior to MRI sessions, participants undergo a 2‐back task (Jaeggi et al., 2010; Owen et al., 2005) as a baseline evaluation of working memory (Figure 8a). Then participants undergo a 2‐back task during the FPS optimization and testing sessions in the MRI. The baseline performance of the 2‐back task is evaluated before the FPS session without concurrent tACS‐fMRI. The cognitive task during the optimization loop is critical to reliably evaluate the tACS effect and successful optimization. In closed‐loop optimization, the effect of tACS with a specific parameter set on the FPS needs to be evaluated online. However, if FPS fluctuates largely regardless of tACS, we cannot judge whether the FPS change is due to the parameter adjustment or spontaneous fluctuation, making parameter optimization impossible. We expect that applying a cognitively demanding task with a strong literature support for engaging frontoparietal network (Cabral‐Calderin et al., 2016; Kuo & Nitsche, 2012; Moisa et al., 2016; Violante et al., 2017; Weinrich et al., 2017b; Williams et al., 2017; Zoefel et al., 2018), that is, the 2‐back task, can reduce spontaneous FPS fluctuations. Even if we still cannot eliminate the fluctuation due to task engagement, those differences could be less than a task‐free paradigm (e.g., resting‐state) (Bogler et al., 2017; Kucyi et al., 2017; Parks & Madden, 2013; Rosenberg et al., 2020).

The performance of the 2‐back task is also used as a behavioral outcome of the tACS effect. The frontoparietal functional connectivity has been implicated in working memory function. Working memory is a key cognitive function that plays a significant role in executive functions and decision making and could be impaired in different mental health disorders, including but not limited to substance use disorders and schizophrenia (Bickel et al., 2011; Brooks et al., 2017; Ieong & Yuan, 2017). Therefore, working memory training has been used in different treatment programs to improve clinical outcomes in different psychiatric and neurologic populations (Brooks et al., 2017; Klingberg et al., 2005; Klingberg et al., 2002). Working memory has been divided into two main processes (Sauseng et al., 2005): (1) executive control, which manages manipulation and retrieval of information from working memory and (2) active maintenance, which maintains the available information. The executive control function is handled by a wide region in the prefrontal cortex, such as the dorsolateral prefrontal cortex (DLPFC), as well as posterior and inferior regions of the prefrontal cortex. Meanwhile, active maintenance is handled mainly within the parietal cortex (Cohen et al., 1997; Prabhakaran et al., 2000). Thus, 2‐back task can be a reasonable outcome for FPS.

The 2‐back task is a well‐established and widely used test with tES in the context of working memory and executive function, while there are also other tasks/contexts that can be used to engage frontoparietal connectivity based on the experimental question/setting (Lau‐Zhu et al., 2017; Pillai et al., 2011). Jaušovec et al. (2014); Violante et al. (2017) reported enhanced working memory performance after stimulating the right frontoparietal sites via tES. We selected the right frontoparietal hemisphere based on previous studies with similar cognitive contexts. We expected that tACS stimulation with optimized parameters would be associated with higher FPS and higher working memory performance than control stimulation.

### 4.3 Online FPS evaluation with real‐time fMRI

Each training and test run includes 15 blocks of tACS stimulation, where each block consists of 20 s stimulation and 10‐s rest period (Figure 8). The 2‐back task is applied continuously irrespective of the tACS block. The task was presented at every 4 s: 2.5 s for presentation and participant response and 1.5 s for the feedback presentation. Each run contained 112 trials of the 2‐back task.

The frontal and parietal regions of interest (ROI) are defined according to the coordinates of the highest electric field on frontal and parietal montage sites obtained from SimNIBS software simulation which firstly were aligned to MNI template (Section 2.5). Those coordinates set as the center of a frontoparietal reference mask in the frontal site (MNI coordinates = [−45, 49, 27], radius = 10 mm) and in the parietal site (MNI coordinates = [−45, −75, 46], radius = 10 mm). The ROI mask defined in the MNI template is warped into the participant anatomical image that is aligned to a reference functional image taken from the resting‐state run (Figure 7a, rsfMRI1). Then the mask is resampled in functional image resolution to create an individual mask for calculating frontoparietal connectivity. This process is done during the first resting‐state run (rsfMRI1) with our fast anatomical image processing system (Misaki et al., 2022). In the optimization runs, fMRI image was processed in real‐time with comprehensive noise reduction (Misaki & Bodurka, 2021), including slice‐timing correction, motion correction, spatial smoothing with 6 mm‐FWHM Gaussian kernel, signal scaling to percent change in each voxel, and regressing out noise components with regressors of high‐pass filtering (Legendre polynomials), motion parameters (three shifts, three rotations), mean white matter and ventricle signals, and physiological noise models with RETROICOR.

**FIGURE 9:**
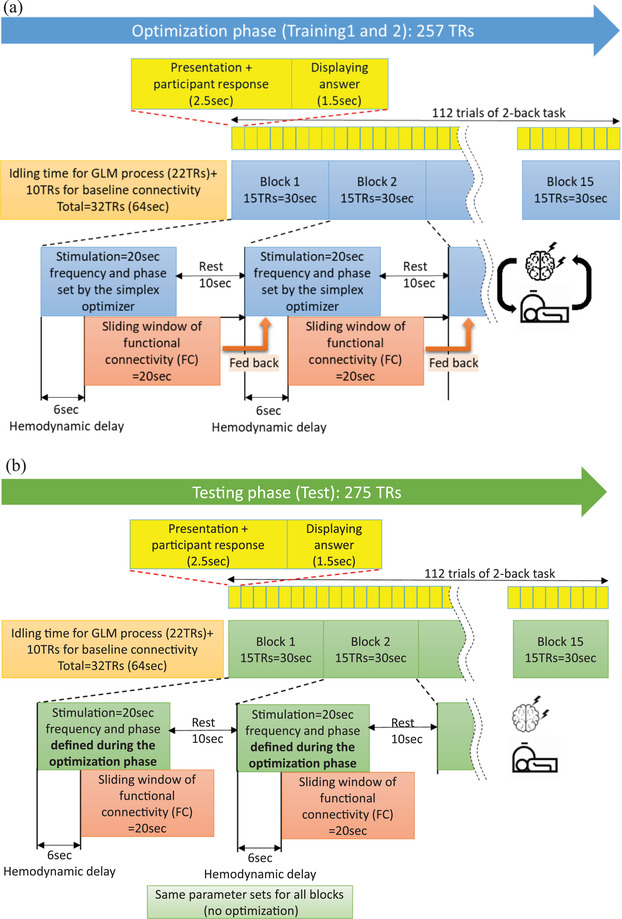
Details of the training and test runs. (a) Training 1 and 2 runs in order to find optimal tACS parameters. Real‐time calculation of functional connectivity (FC) within the frontoparietal network (under F4 and P4) is conducted and is fed back to the optimizer. The optimizer searches through the parameters based on the real‐time FC to maximize its value. The optimizer keeps searching the parameters to maximize FC values in the optimized subject, while the optimizer keeps searching the parameters to minimize FC values in the control subject. (b) Testing run to test the optimal parameters which is found by training runs. During the testing run, subjects in the optimized group will receive the optimized parameters defined to maximize the FC in the training runs, while subjects in the control subject will receive the parameters defined to minimize the FC in the training runs. There are 112 2‐back task trials across runs (4 s for each trial) in order to stabilize the cognitive or subject state changing. The purpose of 2‐back task also for cognitive performance measurement (accuracy of the correct answer).

The functional connectivity between the mean signals of the frontal and parietal ROIs during the 20‐s stimulation block is calculated with the real‐time processed images. The window of connectivity calculation is shifted 6 s, considering the hemodynamic response delay (Figure 9). The connectivity measure is the Pearson correlation with Fisher's r‐to‐z transformation in realtime fMRI processing. This value is used as an outcome of the tACS to optimize the stimulation parameters.

### 4.4 Online tACS parameter optimization

The two tACS parameters, frequency (1–150 Hz) and phase difference (0−359°), are optimized to maximize (or minimize) the FPS for the experimental condition (or for the control condition). Studies proposed that external oscillatory stimulation of cortical regions using various frequencies via tACS can increase internal oscillatory synchronization across brain regions and respective increases in functional connectivity measures (Cabral‐Calderin et al., 2016; Kuo & Nitsche, 2012; Moisa et al., 2016; Violante et al., 2017; Weinrich et al., 2017b; Williams et al., 2017; Zoefel et al., 2018). The flow of information between brain areas may also be flexibly reconfigured through phase synchronization (Akam & Kullmann, 2014; Womelsdorf et al., 2007), and functional connectivity across distant brain regions is also modulated in a phase‐dependent manner (Violante et al., 2017).

One of the significant challenges of the online optimization of tACS parameters are the limited number of trials and unknown parameter–outcome relationship. Because the fMRI functional connectivity calculation requires a certain number of images, the number of testing parameter sets is limited, that is, 30 times in the present protocol (Figure 8). Also, it is not clear how the changes in stimulation parameters are associated with FPS in each individual brain. Thus, we cannot use a gradient‐based optimization method that needs an analytic function of the parameter–outcome relationship.

With these limitations, we consider that the Simplex optimizer with the Nelder‐Mead technique could be a reasonable option (Borggaard, 2019; Mathews & Fink, 2004; Nelder & Mead, 1965; Singer & Nelder, 2009). This algorithm evaluates the outcome of three parameter points at first. Then, the worst point moves to the better parameter direction estimated from the other two points. Repeating this procedure is expected to find the optimal parameter point with enough trials. This method does not require gradient information and could work better than a grid‐search or random search for large parameter space with a limited number of trials (Huang, 2018; Price et al., 2002). The details of Simplex optimizer rules can be found in Nelder and Mead (1965).

Taking the parameters of the initial three trails as close to the optimal point as possible helps the optimizer find the best parameters fast. In Violante et al. (2017), using the same stimulation targets (F4 and P4) and having a similar goal (i.e., to improve cognitive functions measured by the subject's performance in a 2‐back task) as ours, theta band (4–8 Hz) and phase difference = 0° were considered as appropriate parameters. Therefore, we used the center of the theta band (6 Hz) and phase difference = 0° as the center of the equilateral triangle and the edges (6 Hz, 5°), (1 Hz, −3°), and (2 Hz, −3°) are used for the initial three parameters, taking the Hz‐axis and degree‐axis units equal (the 5 unit distance from the center of the triangle to each edge). Since all values for StarStim tACS input must be an integer, we round the decimal parameter values in the optimization.

## 5 STUDY DESIGN

Here we propose a possible study design to examine closed‐loop online tACS‐fMRI optimization performance. We aim to investigate (i) whether the closed‐loop online tACS‐fMRI optimization can find the tACS parameters in terms of enhancing the target functional connectivity during the training runs (the optimization run) and (ii) whether the optimized (i.e., personalized) tACS can influence (i.e., increase) the target functional connectivity during the testing run, compared to a control condition.

**FIGURE 10:**
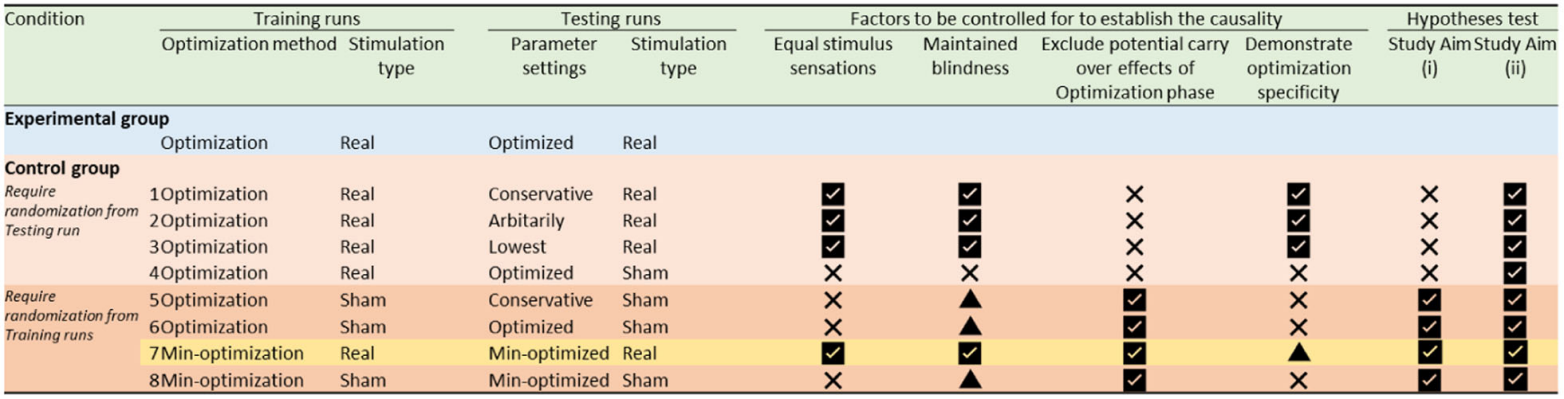
Selection of the control condition and pros and cons. Optimization: Optimizer searches the parameter space to maximize the target functional connectivity (FC). Min‐optimization: Optimizer searches the parameter space to minimize the target FC. Real: each block is composed of 20‐s stimulation and 10 s resting alternatively and repeated for 15 blocks. Sham: each block is composed of 2‐s sham stimulation (i.e. 1‐s ramp up and 1‐s ramp down) and 18 s resting alternatively and repeated for 15 blocks. Conservative: use parameter settings considered to influence target FC based on the literature (e.g., 6 Hz and 0‐degree phase). Arbitrarily: use completely opposite parameter settings with optimized parameters (e.g., frequency × 3.7 modulo 150 ‐ and 180‐degree phase difference). Lowest: using the parameter settings demonstrating the poorest (i.e. lowest) target FC during the training runs. Optimized: use optimized parameter settings defined by the training runs. Min‐optimized: use min‐optimized parameter settings defined by the training runs. Remarks: Control group; 1. Require strong evidence to support the parameter settings. 2. There are no established methods to generate parameter settings completely outside of the optimized parameters. 3. We cannot investigate whether the training runs actually increased target FC compared with other approaches. Study aim (i) cannot be tested. 4. We can only investigate the effect of tACS itself compared with the sham stimulation. Highly doubtful to maintain the blindness since subjects will experience the real stimulation during the training runs. 5. There is a chance that the subject will notice the sham stimulation even if they do not experience the real stimulation. 6. There is a chance that the subject will notice the sham stimulation even if they do not experience the real stimulation. 7. We can conclude that the group difference is specified by the optimization method because subjects will experience a min‐optimization approach during the training runs, which may affect the target FC. Therefore, we can test both study aims (i) and (ii). 8. There is a chance that the subject will notice the sham stimulation even if they do not experience the real stimulation.

The study aims cannot be tested without a control condition since we cannot exclude non‐specific changes in functional connectivity (e.g., due to boredom, habituation with MRI environment, alertness, etc.). We propose and summarize all possible control conditions for this study in Figure 10. To test study aims (i) and (ii), we decided to apply the control condition described in Figure 10, condition no. 7. In short, during the testing run, a participant in the experimental condition will receive tACS with the parameters that maximize FPS in the training runs, while a participant in the control group will receive tACS with the parameters that minimize FPS. Future studies might like to try other control conditions based on their study questions.

### 5.1 Procedures

Figure 8 shows the procedures of the experiment for the experimental (optimized; maximize FPS) and the control (non‐optimized; minimize FPS) groups. For the two training FPS sessions, participants are randomly assigned to either an optimized (experimental) group or a control group. For the optimized group (experimental group), the optimizer searches the tACS parameters that can achieve the highest FPS, while for the control group, the optimizer searches the tACS parameters that can achieve the lowest FPS during the training runs. Then participants undergo a testing scan, in which they are stimulated with the optimized (in the experimental group) or control parameters (in the control group) during the training runs. The testing run tests the optimized parameters’ ability to directionally modulate the FPS. The testing run is similar to the training runs, which is divided into 15 blocks (Figure 9b), while the parameters are fixed to that obtained in the training 1 and 2 runs. Resting‐state scans (rsfMRI) are applied before and after the FPS optimization sessions and after the test session. Each rsfMRI scan lasts 6 min 50 s.

A small but growing body of evidence suggests the washout period should be at least half of the stimulation period. For example, Beeli et al. (2008) used 3.5 min rest between different stimulation conditions as their washout period (each condition lasted for 5 min). Considering the whole training run (17 min 8 sec) as a stimulation period, about 7‐min intervals with a rest scan between the training and test scans could be safe for settling the aftereffects (Nitsche & Paulus, 2001; Shafi et al., 2012). Then, in the test scan, we would be able to evaluate the stimulation effect with an optimized parameter set apart from the aftereffects.

### 5.2 Hypotheses and expected outcomes

The hypotheses and expected outcomes of the study with this protocol are the following.

**Hypothesis 1**: Regarding the first study aim, we hypothesize that a participant in the experimental condition will show increased frontoparietal functional connectivity, while the participant in the control condition will show decreased frontoparietal functional connectivity on the course of training1 and 2. We looked for the best tES parameters within each subject's training runs (intraindividual variability) without any comparison to other subjects.
**Hypothesis 2**: Regarding the second study aim, we hypothesize that the optimized (i.e., personalized) tACS parameter settings will increase the fMRI connectivity between the tACS targets (under the electrodes of F4–P4) during the testing run for the experimental condition while the control tES parameters will decrease the connectivity for the control condition.
**Hypothesis 3**: The experimental group will show improvement in the accuracy on the 2‐back task from training to the testing run, compared to the control group.

## 6 PRELIMINARY RESULTS

We perform a pilot experiment for two healthy participants to confirm the feasibility of the protocol implementation. The study is approved by the Western Internal Review Board (WIRB #20200192) and the participants gave informed consent. The experiment is performed according to the protocol presented in Section 4.

**FIGURE 11:**
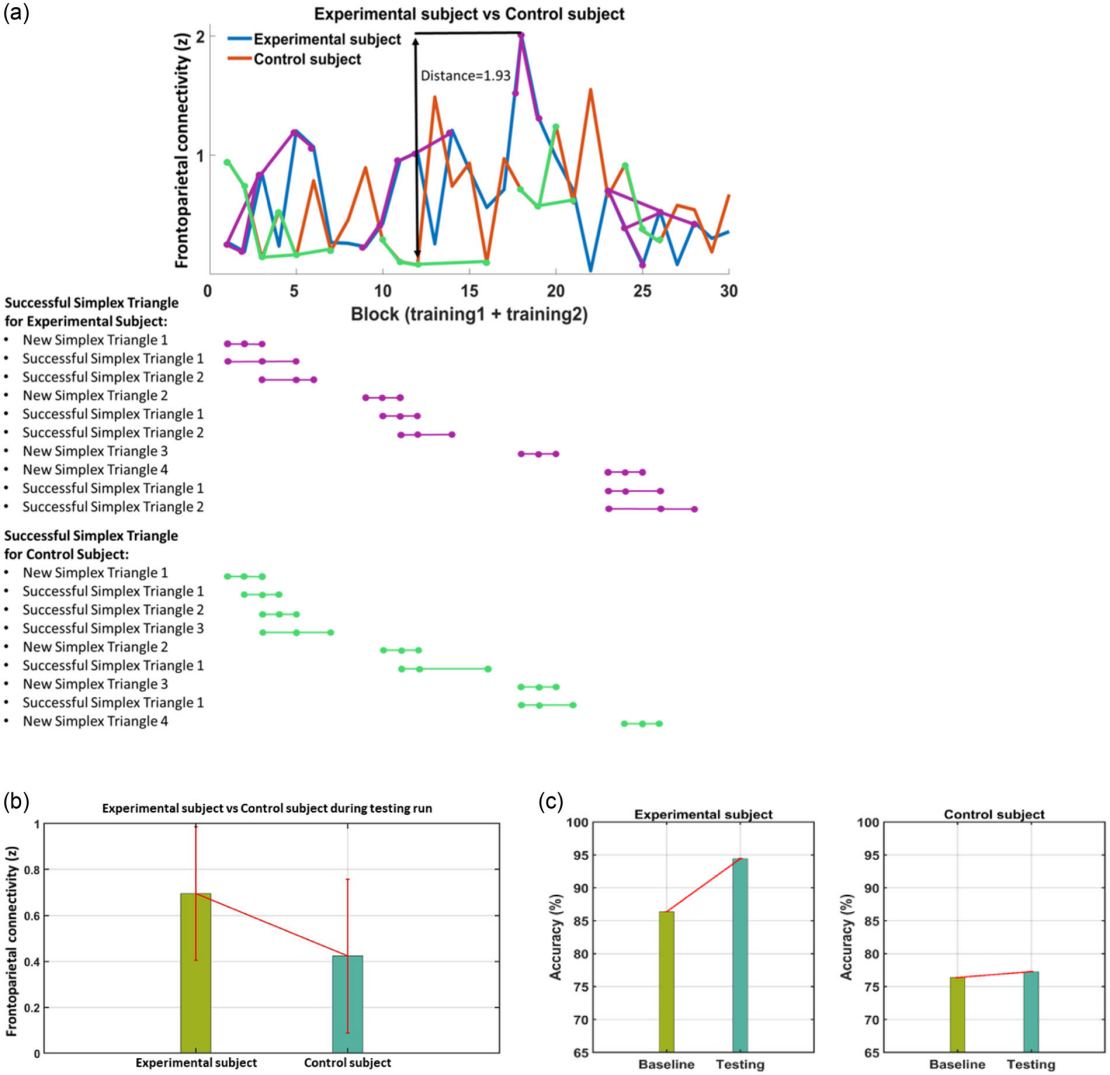
Sample data from the closed‐loop tACS‐fMRI protocol with two participants from the experimental and control conditions. (a) Blocks of training 1 and 2 for each subject. There are 30 blocks for training 1 and 2. During training 1 and 2, blocks can be divided into two; successful or failed blocks. Failed attempts could not be counted as a path to highest or lowest connectivity. The successful blocks track to find the highest connectivity in the experimental subject are magenta‐circle‐line; meanwhile, the successful blocks track to find the lowest connectivity on the control subject are green‐circle‐line. During training 1 and 2, the Simplex optimizer will create four new sequential of the Simplex triangles. At the end of the training, we chose the best sequential of the Simplex triangle that maximizes/minimizes the online frontoparietal connectivity either for an experimental or a control subject. For the experimental subject, the third sequential of the Simplex triangle at the second block with tES parameter frequency = 1 Hz, and phase = −1^o^ was selected, since it achieved the highest online frontoparietal connectivity = 2.01. For the control subject, the second sequential of the Simplex triangle at the third block with tES parameter frequency = 3 Hz, and phase = −6^o^ was selected since it achieved the lowest online frontoparietal connectivity = 0.08. The Euclidian distance of the maximum or minimum online frontoparietal connectivity response between experimental and control subjects was 2.01–0.08 = 1.93. (b) Measure of task‐based online frontoparietal connectivity; (c) Behavioral outcomes of the protocol. There is no statistical/hypothesis testing intended for this figure. This is just to depict the structure of the data and outcomes.

Figure 11a shows the pilot/feasibility data for visualizing changes in the target functional connectivity during the training runs for an experimental (44 years of age, female) and a control (46 years, female) participant, respectively. As seen in the plot, the search for optimal parameters to get the highest or lowest frontoparietal connectivity did not show a monotonic increase or decrease of the connectivity, suggesting that the outcome (FPS) hyperplane in the parameter space is highly unsmooth. In the Simplex optimization process, a parameter point that failed to increase (or decrease in the control) connectivity was discarded and took another point by changing the step size or direction. The successful blocks for the experimental subject are illustrated with a magenta‐circle‐line in Figure 11a. Meanwhile, the successful blocks for the control subject are illustrated with a green‐circle‐line in Figure 11a. When the Simplex optimizer failed to achieve the successful block two times, a new Simplex triangle was created.

Although monotonic increase/decrease could not be made during the search, the connectivity for the experimental participant was higher on average than for the control participant, indicating that the Simplex search approached the parameters that increased the connectivity in the experimental participant. Even if the global optimal point could not be found with the limited number of trials, better parameter sets than the initial point could be obtained during the search.

Figure 11b shows the mean frontoparietal connectivity of the blocks in the test run. For the experimental participant, our algorithm selected frequency = 1 Hz and phase difference = −1°, which achieved the highest connectivity in the training runs. For the control participant, our algorithm selected frequency = 3 Hz and phase difference = −6°, which achieved the lowest connectivity in training runs. The mean connectivity for the experimental participant was higher compared to the control participant (experimental subject: mean = 0.70, SD = 0.29; control subject: mean = 0.42, SD = 0.33; t(28) = 2.38, *p* = .025).

We are aware that z = ∼2.0 for the experimental subject in Figure 11a seems too high for natural connectivity, suggesting potential head motion or noise effect. Nevertheless, in the testing run, we observed that the experimental subject showed higher connectivity with the optimized parameters defined by the training runs compared to the control participant (Figure 11b). This result demonstrates that the optimization could approach better parameters even with potential noises. In the future studies, censoring time points with excessive motion or global signal regression (Misaki & Bodurka, 2021; Weiss et al., 2020) may further improve real‐time functional connectivity evaluation.

We also examined the working memory (2‐back task) performance (Figure 11c) and found that the experimental participant showed improved accuracy during the testing run compared to the baseline (before the tACS optimization training), while the control participant did not show any improvement. Accuracy improvement was 8.08% for the experimental participant and 0.91% for the control participant. These results accord with our hypotheses, while no statistical testing or inferencing is intended.

While only a single participant result for each condition cannot prove our hypotheses, this pilot experiment confirms at least that our proposed protocol could be implemented in practice and worked as designed.

## 7 CONCLUSION

**FIGURE 12:**
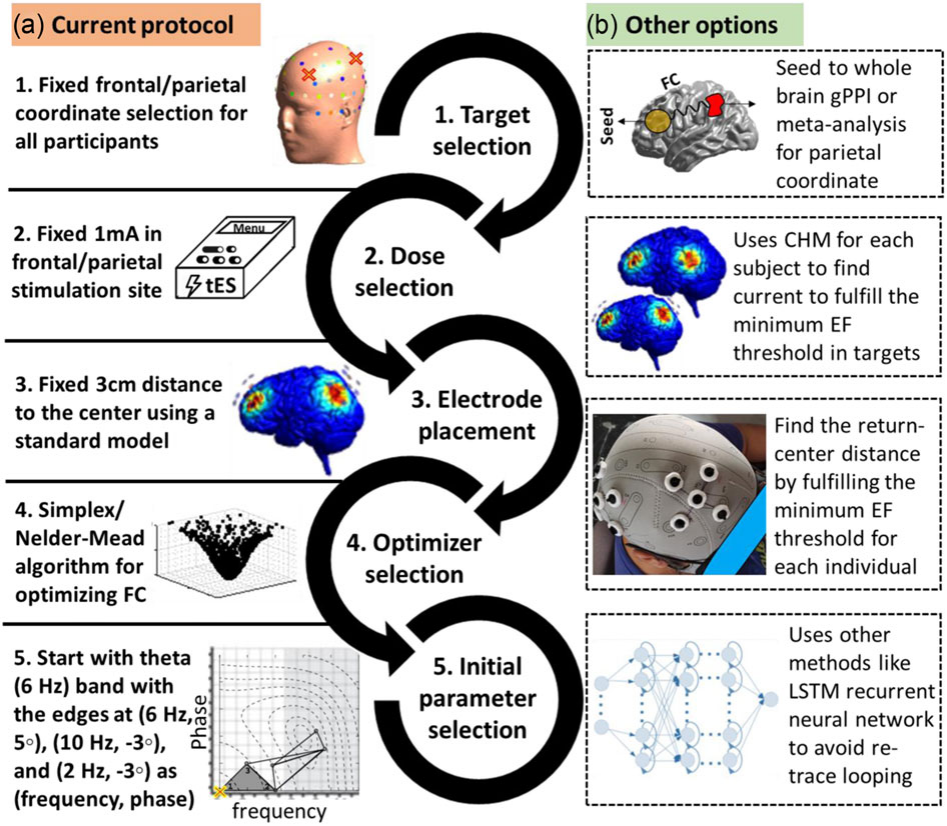
Current protocol and other potential options in designing a closed‐loop tES‐fMRI system. (a) Current protocol. In the current proposed pipeline, there are five main steps: (1) fixed frontal and parietal coordinates were used for all participants based on the 10‐10 EEG standard system and previous studies with a similar purpose (working memory enhancement), (2) Fixed 1‐mA peak‐to‐peak current intensity is used in both frontal and parietal sites for all subjects refer to the previous study and by considering safety inside the scanner. (3) Fixed 3 cm between electrode distance (between the center and peripheral electrodes) is used based on electric field calculations to have a satisfying focality in the targeted brain region. (4) Simplex/Nelder‐Mead optimizer is used for finding optimized stimulation parameters because of fast and simple computation and fairly robust searching algorithm. (5) Starting point for the Simplex optimizer is in the theta band with the edges at (frequency in Hz, phase difference in degree): (6, 5), (10, −3), and (2, −3) to have a faster search in the optimization. (6) Defining training and testing runs for stimulation protocols. Training 1 and 2: 20 s Stim, 10 s no‐Stim, and repeated 15 times. There are 7 min rsfMRI time to washout aftereffects stim between training 1 and training 2 and testing. Experimental group will find the best tES parameters to increase functional connectivity (FC), otherwise the Control. (b) Other options: There are many other options for the decisions made in the current protocol. For example, seed to whole‐brain analysis can be performed for finding connected regions, computational head models (CHMs) can be used for determining optimized current intensity for each individual based on personalized brain structure to fulfill the minimum EF threshold in order to engage the brain target activity. Between electrode distance can be determined based on personalized skull shape and simulated electric fields, other optimization algorithms like Long‐Short Term Memory (LSTM) network or Bayesian optimization can be used for finding optimized stimulation parameters. It would be possible to optimize timing in application of electrical stimulation, data collection or task‐fMRI task design. *Abbreviations*: CHM, computational head model; EF, electric field; LSTM, long short‐term memory.

The summary of the current protocol and other potential options in designing a closed‐loop tES‐fMRI system is illustrated in Figure 12. We introduced an online frontoparietal stimulation closed‐loop tACS‐fMRI protocol. We described the concurrent tACS‐fMRI equipment settings, including HD electrodes and montages, online connectivity evaluation with real‐time fMRI, and the optimization algorithm. Our simulation analysis shows that the focality of electric current stimulation can be obtained under each frontal and parietal site during different phase conditions. Furthermore, by the specific return electrode placement, we can reduce the shunt effect of different phase stimulations to minimal values (stimulated area only increases 4.12% in the antiphase stimulation compared to the in‐phase stimulation). We conducted a safety/noise test for this proposed protocol using watermelon and a single human subject, and confirmed that our concurrent tACS‐fMRI setting does not cause any adverse heating effects or image artifacts.

Also, we suggested the Simplex optimizer (Nelder‐Mead technique) as a simple optimization algorithm with a light computational burden, which is suitable for real‐time closed‐loop experimental settings with a limited number of parameter search steps. With a task requiring the cognitive load (instead of resting‐state), we could expect less fluctuation in the frontoparietal functional connectivity. Utilizing a cognitive task (e.g., 2‐back task) to stabilize functional connectivity during the course of stimulation would help the Simplex optimizer search parameter space efficiently.

While the present protocol optimized the tACS frequency and phase difference parameters, optimizing the stimulation coordinates (electrodes positions) may further improve the efficacy of the stimulation. Although the stimulation coordinates in the present protocol were determined based on previous tES studies (Jaušovec et al., 2014; Violante et al., 2017), a newly developed method attempted to optimize the stimulation coordinates utilizing fMRI (Soleimani et al., 2021) can be incorporated in the future studies.

We confirmed the present protocol could be implemented in practice and worked as expected for a pilot participant. In the future study, we will run the experiment with this protocol for more participants to examine the efficacy of the personalized tACS intervention on the frontoparietal connectivity and its functional benefit in working memory performance.

## CONFLICT OF INTEREST

The authors declare that the research is conducted in the absence of any commercial or financial relationships that could be construed as a potential conflict of interest.

## AUTHOR CONTRIBUTIONS

Beni Mulyana, Hamed Ekhtiari, Jerzy Bodurka, Masaya Misaki, Aki Tsuchiyagaito, and Rayus Kuplicki designed the study. Beni Mulyana, Aki Tsuchiyagaito, and Jared Smith collected the data. Beni Mulyana performed simulations and data analyses under Hamed Ekhtiari, Masaya Misaki, Jerzy Bodurka, Samuel Cheng, and Rayus Kuplicki supervision. Beni Mulyana wrote the paper with input from Hamed Ekhtiari, Til Ole Bergman, Duke Shereen, Masaya Misaki, Rayus Kuplicki, Ghazaleh Soleimani, Aki Tsuchiyagaito, Ashkan Rashedi, Samuel Cheng, Jerzy Bodurka, and Martin P. Paulus. All authors (Beni Mulyana, Hamed Ekhtiari, Til Ole Bergman, Duke Shereen, Masaya Misaki, Rayus Kuplicki, Ghazaleh Soleimani, Aki Tsuchiyagaito, Jared Smith, Ashkan Rashedi, Samuel Cheng, Jerzy Bodurka, and Martin P. Paulus) contributed to manuscript preparation. All authors (Beni Mulyana, Hamed Ekhtiari, Til Ole Bergman, Duke Shereen, Masaya Misaki, Rayus Kuplicki, Ghazaleh Soleimani, Aki Tsuchiyagaito, Jared Smith, Ashkan Rashedi, Samuel Cheng, Jerzy Bodurka, and Martin P. Paulus) agreed on the final manuscript before submission.

### PEER REVIEW

The peer review history for this article is available at https://publons.com/publon/10.1002/brb3.2667

## Supporting information


**Figure S1**. The electrodes from the sagittal side view. d1, d2, d3, d4 and Ɵ1, Ɵ2, Ɵ3, Ɵ4 have two points of view; [1] distance and skewed angle from the center electrode to return electrodes in frontal (F4) and parietal (P4) sites which is related to point A, [2] distance and skewed angle from the center in between sites to each electrode (F4, P4 and their returns) in frontal (F4) and parietal (P4) sites which is related to point B. (a) In the in‐phase condition, the electric field at point p is dominant from electrodes above its point p; (b) In the in‐phase condition, the electric field at point p position between frontal and parietal is ≈ 0; C) The electric field generated by electrodes in anti‐phase condition. The electric field at point p positiosn between frontal and parietal is ≠ 0. r1 and r2 are the distance from the scalp to point p inside the brain.
**Supporting Information A**. Electric field derivation in‐phase and anti‐phase condition
**Supporting Information B**. MRI Artifacts, fMRI Noise Testing Method

## Data Availability

The data and analysis codes that support the findings of this study are available from the corresponding author upon reasonable request.
