# Supplementary material for: Online closed‐loop real‐time tES‐fMRI for brain modulation: A technical report
Source: Brain Behav. 2022 Sep 22;12(10):e2667. doi: 10.1002/brb3.2667 (PMC9575607; doi:10.1002/brb3.2667)
Supplement: Supplementary file 1 — Figure S1. The electrodes from the sagittal side view. d1, d2, d3, d4 and Ɵ1, Ɵ2, Ɵ3, Ɵ4 have two points of view; [1] distance and skewed angle from the center electrode to return electrodes in frontal (F4) and parietal (P4) sites which is related to point A, [2] distance and skewed angle from the center in between sites to each electrode (F4, P4 and their returns) in frontal (F4) and parietal (P4) sites which is related to point B. (a) In the in‐phase condition, the electric field at point p is dominant from electrodes above its point p; (b) In the in‐phase condition, the electric field at point p position between frontal and parietal is ≈ 0; C) The electric field generated by electrodes in anti‐phase condition. The electric field at point p positiosn between frontal and parietal is ≠ 0. r1 and r2 are the distance from the scalp to point p inside the brain. Supporting Information A. Electric field derivation in‐phase and anti‐phase condition Supporting Information B. MRI Artifacts, fMRI Noise Testing Method [file BRB3-12-e2667-s001.docx]

**Supplementary Materials**

**Online Closed-Loop Real-Time tES-fMRI for Brain Modulation: A Technical Report**

Beni Mulyana^1,2^, Aki Tsuchiyagaito^1^, Masaya Misaki^1^, Rayus Kuplicki^1^, Jared Smith^1^, Ghazaleh Soleimani^5, 6^, Ashkan Rashedi^1^, Duke Shereen^3^, Til Ole Bergman^7,8^, Samuel Cheng^2^, Martin Paulus^1^, Jerzy Bodurka^1,4^ and Hamed Ekhtiari^1,9^

1. Laureate Institute for Brain Research, Tulsa, OK, USA
2. Electrical and Computer Engineering, University of Oklahoma, Tulsa, Oklahoma, USA
3. The Graduate Center of the City University of New York, New York, NY, USA
4. Stephenson School of Biomedical Engineering, University of Oklahoma, Norman, OK, USA
5. Amirkabir University of Technology, Tehran, Iran
6. Iranian National Center for Addiction Studies, Tehran, Iran
7. Neuroimaging Center (NIC), University Medical Center of the Johannes Gutenberg University Mainz, Germany
8. Leibniz Institute for Resilience Research (LIR), Mainz, Germany
9. Department of Psychiatry and Behavioral Sciences, University of Minnesota, MN, USA

Corresponding Author:

Hamed Ekhtiari, MD, PhD

Laureate Institute for Brain Research

6655 S Yale Ave, Tulsa, OK 74136-3326

Phone: 918 502 5120; email: [hekhtiari@laureateinstitute.org](mailto:hekhtiari@laureateinstitute.org)

ORCID ID: <https://orcid.org/0000-0001-6902-8798>

## Electric field derivation in-phase and anti-phase condition

F4 and P4 electrodes in the 10-20 system are the centers of stimulation, with the current function of $F4=A\times\sin\left( 2\pi\times freq_{F4}\times t + phase_{F4} \right)$, and of $P4=A\times\sin\left( 2\pi\times freq_{P4}\times t + phase_{P4} \right)$. To reduce space complexity in finding optimal parameters and to reduce training time, the electric current $'A^{'}$ will not become a parameter that will be searched by the optimizer, but it will be fixed to 1 mA-peak value. The current function of each of the F4 returning-electrodes is such that: $\frac{A}{4}\times\sin\left( 2\pi\times freq_{F4}\times t+phase_{F4}+{180}^{o} \right)$. The current is divided by 4 and a phase of 180^o^ is added in order to fulfill Kirchhoff’s law (Labate & Matekovits, 2016; Paul, 2001). Likewise, the electric current function on each of the P4 returning-electrodes is that: $\frac{A}{4}\times\sin\left( 2\pi\times freq_{P4}\times t+phase_{P4}+{180}^{o} \right)$. To calculate the electric field on the cortex, we use the method described by Saturnino et al., 2017. Once the electric current is applied through electrode on the scalp, then the electric field at position p inside the head and at time point t is determined by the product of the spatial component ***E***(p) and the time course of the electric current *I*(t) injected into the active channel (equation 1):

***E***(p,t) = ***E***(p) × *I*(t) [1]

Because we have 10 electrodes which emit the current mentioned above, the total electric field at the point p as described in equation 2:

$\boldsymbol{E}\left( p,t \right)= \sum_{i=1}^{10} \boldsymbol{E}_{i}\left( p \right)I_{i}\left( t \right)$ [2]

For a simpler analysis, we ignore the head curvature and draw the surface of the head in which the electrodes are positioned as a plane surface, as shown in Figures S1B and C, and the electrodes side view as shown in Figure S1A (with where the peripheral electrodes aligned in the direction of view and occluding each other being and combined into one electrode). In equation 1, the spatial component ***E***(p) is inversely proportional to conductivity ($\kappa$) at point p and the square of the distance ($r$) between the electrode to point p or $\left( \frac{1}{{\kappa r}^{2}} \right)$. If the $phase_{F4}$ is equal to $phase_{P4}$, termed *in-phase* condition, then the electric field at p under P4 on the y-axis, and at time $t_{0}$ using equation 2 can be written as equation 3:

$\boldsymbol{E}\left( p,t_{0} \right)=\boldsymbol{E}_{1}\left( p \right)I_{1}cosƟ_{1}-\boldsymbol{E}_{2}\left( p \right)I_{2}+\boldsymbol{E}_{3}{\left( p \right)I}_{3}cosƟ_{1}+\boldsymbol{E}_{4}\left( p \right)I_{4}cosƟ_{2}-\boldsymbol{E}_{5}{\left( p \right)I}_{5}cosƟ_{3}+\boldsymbol{E}_{6}\left( p \right)I_{6}cosƟ_{4}$we know, $\boldsymbol{E}_{i}\left( p \right)$ is proportional to $\left( \frac{1}{{\kappa r}^{2}} \right)$, for simplification, we assume κ =1 at point p, and $I_{1}=I_{3}=0.5I_{2} , I_{4}=I_{6}=0.5I_{5} , and I_{2}=I_{5}=I_{o}$. If $r_{2}$ is the distance from electrode P4 to point p, then equation 3 can be written as equation 4:

$\boldsymbol{E}\left( p,t_{0} \right)\approx\frac{{0.5I}_{o}}{d_{1}^{2}}{cosƟ_{1}{sin}^{2}Ɵ}_{1}-\frac{I_{o}}{r_{2}^{2}}+ \frac{{0.5I}_{o}}{d_{1}^{2}}{cosƟ_{1}{sin}^{2}Ɵ}_{1}+ \frac{{0.5I}_{o}}{d_{2}^{2}}{cosƟ_{2}{sin}^{2}Ɵ}_{2}- \frac{I_{o}}{d_{3}^{2}}{cosƟ_{3}{sin}^{2}Ɵ}_{3}+ \frac{{0.5I}_{o}}{d_{4}^{2}}{cosƟ_{4}{sin}^{2}Ɵ}_{4}$ [4]

Note components: $\frac{{0.5I}_{o}}{d_{2}^{2}}{cosƟ_{2}{sin}^{2}Ɵ}_{2}- \frac{I_{o}}{d_{3}^{2}}{cosƟ_{3}{sin}^{2}Ɵ}_{3}+ \frac{{0.5I}_{o}}{d_{4}^{2}}{cosƟ_{4}{sin}^{2}Ɵ}_{4}$can be neglected because they are closed to zero since $Ɵ_{2}\approxƟ_{3}{\approxƟ}_{4} \approx9$0^o^ or $r_{2}$ is small, then $cosƟ_{2}\approx cosƟ_{3}\approx cosƟ_{4}\approx0$.

Therefore, $\boldsymbol{E}\left( p,t_{0} \right)\approx\frac{{0.5I}_{o}}{d_{1}^{2}}{cosƟ_{1}{sin}^{2}Ɵ}_{1}-\frac{I_{o}}{r_{2}^{2}}+ \frac{{0.5I}_{o}}{d_{1}^{2}}{cosƟ_{1}{sin}^{2}Ɵ}_{1}$and is only influenced by electrodes above the cortex. Likewise, when we analyze along the x-axis, the electric field in in-phase condition will be dominant from electrodes above the cortex surface. If we put the p position in between frontal and parietal (Figure S1B), the electric field on the y-axis in that point is:

$$\boldsymbol{E}\left( p,t_{0} \right)\approx\frac{{0.5I}_{o}}{d_{1}^{2}}{{{cosƟ}_{1}sin}^{2}Ɵ}_{1}- \frac{I_{o}}{d_{2}^{2}}{{{cosƟ}_{2}sin}^{2}Ɵ}_{2}+ \frac{{0.5I}_{o}}{d_{3}^{2}}{{{cosƟ}_{3}sin}^{2}Ɵ}_{3}+ \frac{{0.5I}_{o}}{d_{1}^{2}}{{{cosƟ}_{1}sin}^{2}Ɵ}_{1}- \frac{I_{o}}{d_{2}^{2}}{{{cosƟ}_{2}sin}^{2}Ɵ}_{2}+ \frac{{0.5I}_{o}}{d_{3}^{2}}{{{cosƟ}_{3}sin}^{2}Ɵ}_{3}$$

or

$\boldsymbol{E}\left( p,t_{0} \right)\approx\frac{I_{o}}{d_{1}^{2}}{{{cosƟ}_{1}sin}^{2}Ɵ}_{1}- \frac{{2I}_{o}}{d_{2}^{2}}{{{cosƟ}_{2}sin}^{2}Ɵ}_{2}+ \frac{I_{o}}{d_{3}^{2}}{{{cosƟ}_{3}sin}^{2}Ɵ}_{3}$ [5]

Once again if$Ɵ_{1}\approxƟ_{2}{\approxƟ}_{3} \approx9$0^o^ or if $r_{2}$ is small, then $\boldsymbol{E}\left( p,t_{0} \right)\approx0$. The electric field along the x-axis is also 0 since all x-component are cancelled each other. Thus, in the in-phase condition, there is no electric field in any volume between frontal and parietal electrodes. Therefore, it can be concluded that the electric field on the in-phase condition from our montage will appear under frontal and parietal electrodes but will not appear in between under frontal and parietal electrodes. Then, what is the electric field in between sites if we change into anti-phase condition? It is anti-phase in the condition when $phase_{F4}$ and $phase_{P4}$ differ by 180^o^. The electric field generated from every electrode at the time $t_{0}$ for anti-phase is illustrated in Figure S1C. From Figure S1C, we derive the electric field at point p and time $t_{0}$ along x-axis such that:

$$\boldsymbol{E}\left( p,t_{0} \right)\approx- \frac{{0.5I}_{o}}{d_{1}^{2}}{{{sinƟ}_{1}sin}^{2}Ɵ}_{1}+ \frac{I_{o}}{d_{2}^{2}}{{{sinƟ}_{2}sin}^{2}Ɵ}_{2}- \frac{{0.5I}_{o}}{d_{3}^{2}}{{{sinƟ}_{3}sin}^{2}Ɵ}_{3} - \frac{{0.5I}_{o}}{d_{1}^{2}}{{{sinƟ}_{1}sin}^{2}Ɵ}_{1}+ \frac{I_{o}}{d_{2}^{2}}{{{sinƟ}_{2}sin}^{2}Ɵ}_{2}- \frac{{0.5I}_{o}}{d_{3}^{2}}{{{sinƟ}_{3}sin}^{2}Ɵ}_{3}$$

or

$\boldsymbol{E}\left( p,t_{0} \right)\approx- \frac{I_{o}}{d_{1}^{2}}{{{sinƟ}_{1}sin}^{2}Ɵ}_{1}+ \frac{2I_{o}}{d_{2}^{2}}{{{sinƟ}_{2}sin}^{2}Ɵ}_{2}- \frac{I_{o}}{d_{3}^{2}}{{{sinƟ}_{3}sin}^{2}Ɵ}_{3}$ [6]

If $Ɵ_{1}\approxƟ_{2}{\approxƟ}_{3}\approx9$0^o^ or $r_{2}$ is small, then $\boldsymbol{E}\left( p,t_{0} \right)\approx- \frac{I_{o}}{d_{1}^{2}}+ \frac{2I_{o}}{d_{2}^{2}}- \frac{I_{o}}{d_{3}^{2}}$ , and $\left| - \frac{I_{o}}{d_{1}^{2}}+ \frac{2I_{o}}{d_{2}^{2}}- \frac{I_{o}}{d_{3}^{2}} \right|$could be larger than 0 if $d_{1}\ll d_{2}<d_{3} ,or d_{1}≉d_{2}≉d_{3}$. The electric field along y-axis = 0, caused by every component is cancelled each other.

## MRI Artifacts, fMRI Noise Testing Method

We use the same tACS stimulation device (Starstim R32; Neuroelectrics Barcelona SLU; Spain) inside the MRI (3T MRI scanner (Discovery MR750; GE Healthcare Systems, Milwaukee, WI) with an 8-channel receive-only head coil). Single-shot gradient-recalled echo-planner imaging (EPI) with sensitivity encoding (SENSE) is used for the scans with the parameters of FOV = 240 × 240 mm, matrix = 96 × 96 reconstructed into 128 × 128, SENSE acceleration factor R = 2, 45 axial slices with slice thickness = 2.9 mm, TR/TE = 2/0.025seconds, flip angle = 90^o^. The tACS montage is the same as described in section 2.1, which used 10 HD electrodes with 4 × 1 ring montage at two sites (F4 and P4 are active electrodes). F4 and P4 electrodes are selected as the main nodes of the frontoparietal network (1mA, 6Hz, 0^o^ phase difference; Figures 4 and 5), and eight electrodes are used as return electrodes surrounding F4 and P4 with coordinates are described in section 2.1. We use three scanning protocols for different goals. The first scan is obtained without radio frequency (RF) to evaluate the impact of tACS on the EPI k-space during no-RF excitation or only noise to draw EPI images. The second is obtained with RF excitation to evaluate the impact of tACS on voxel-wise EPI images and temporal signal to noise ratio (TSNR). And the third one is to confirm tACS-fMRI safety. We used a watermelon phantom for the first and second scans to avoid the effect of a neural activation signal modulated by tACS stimulation. The stimulation block in the first and second scan is a 20-seconds tACS stimulation ON and following by a 10-seconds no-stimulation, then the block is repeated 15 times with a 12-seconds OFF block at the beginning. The initial 12-seconds of data are excluded from the analysis to ensure a steady-state fMRI signal. Without-RF EPI scanning data on the first scan has dimension [128 128 45] and time course = 225TRs ($10\times15=150TRs$ from Stim ON, and $5\times15=75TRs$ from Stim OFF). Data is transformed by two-dimensional fast Fourier transform (2D FFT) to obtain k-space data. Since this data is collected without-RF excitation, we can evaluate the tACS influence on the MRI system noise in the receiving signal free from the sample signal, and without having a flip angle to synchronize proton magnetization phases. Therefore, phase-encoding will be encoded randomly from 0^o^ until 360^o^, asynchronous with tACS, ensuring that the noise amplitude in the phase encoding direction collapses once averaged, leaving the data frequency-encoding and z-slice selected along the time course (dimension: [128 45 225]). Next, ON and OFF stimulation data are separated, and a t-test is performed between ON/OFF conditions for each frequency encoding (kx-direction) and z-slice selection. All corresponding p-values are corrected for multiple hypothesis comparisons testing using the Benjamini and Hochberg procedure for False Discovery Rate (FDR) discovery (Benjamini & Hochberg, 1995). For the voxel-wise analysis in the second scan, we performed GLM analysis on the image time-course data using 3dDeconvolve in AFNI (<https://afni.nimh.nih.gov/>). The regressors included a boxcar time-series for the ON period and 3rd-order Legendre polynomials to remove the low-frequency fluctuation. We also compared mean value of time series in each voxel within the ROIs (F4 and P4) and its SD for the ON and OFF period. For TSNR analysis, it also uses EPI data images with-RF scan. First, ON and OFF stimulation data of EPI data images are separated. Then TSNR is calculated for each ON and OFF stimulation data using 3dTstat to create voxel-wise TSNR.

For the fMRI safety evaluation in the third protocol, we performed a concurrent tACS-fMRI scan for a healthy female volunteer (age 38 years) to measure temperature change on the scalp under the electrodes due to concurrent tACS stimulation during fMRI. The temperature is obtained by placing MRI compatible temperature sensors (Biopac TSD202A and Biopac SKT100C, sensitivity = 100 micro °C, sample rate = 200points/seconds) under the electrodes (P4 and F4). We used the same montage (Figures 4 and 5), fMRI parameters, and tACS parameters as in the first and second scans. We collected a baseline temperature for 2 minutes before the scanning and stimulation. Then, 2-minutes ON and OFF blocks are repeated three times to see the tACS effect in a long time period. The temperature difference between the ON and OFF periods is tested with z statistics. This study was conducted in accordance with the Declaration of Helsinki and all methods were carried out in accordance with relevant guidelines and IRB approval.


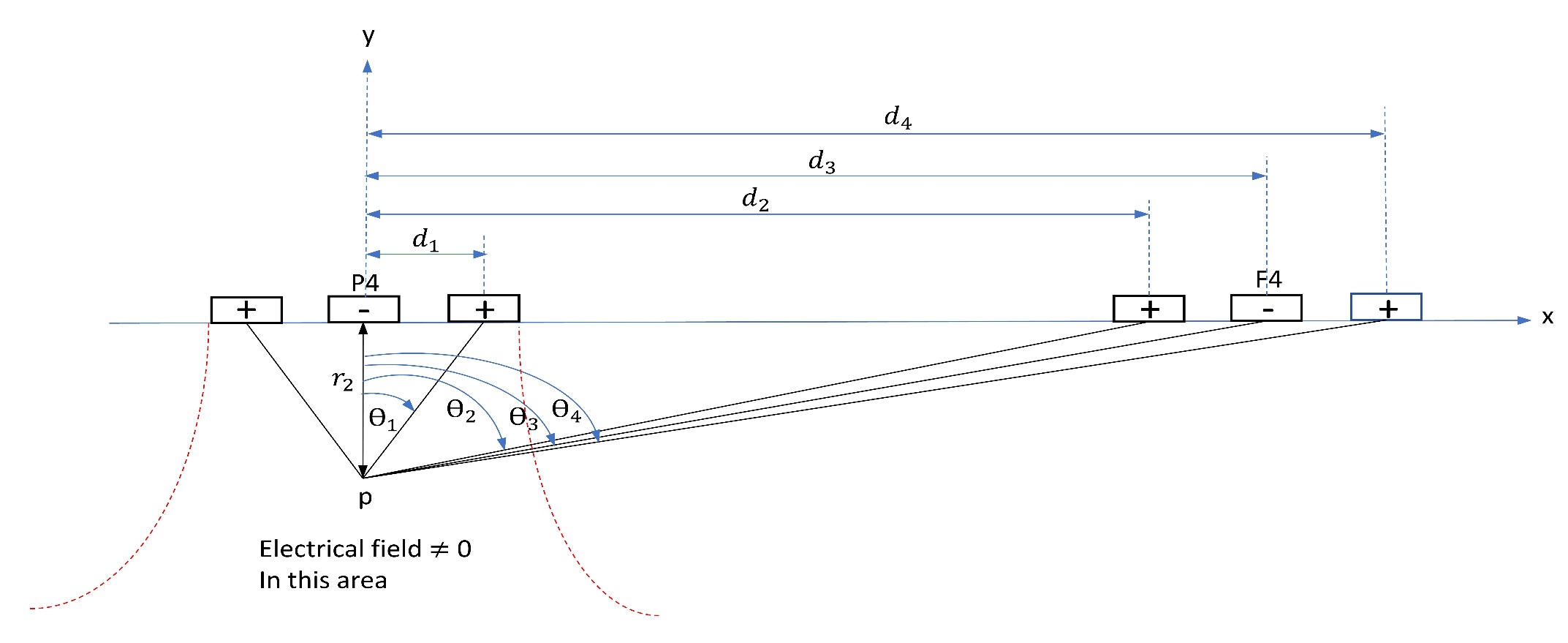


**A**


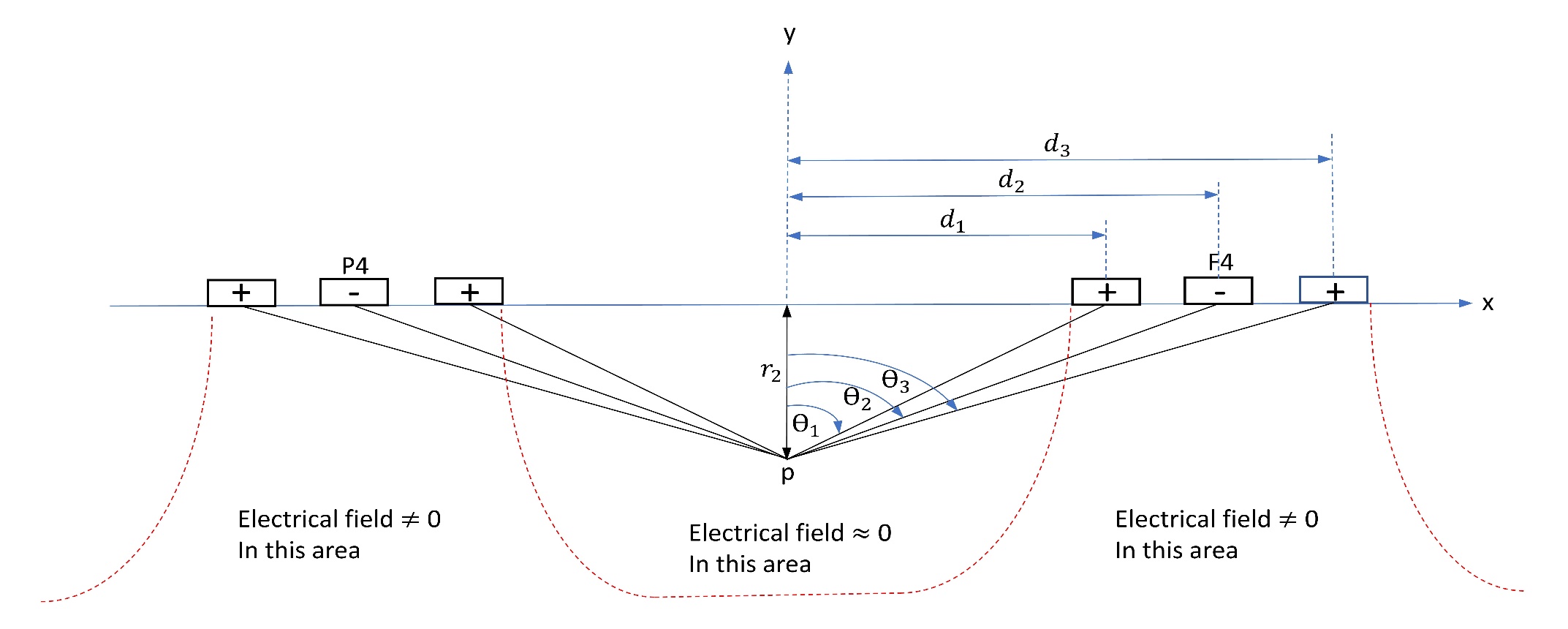


**B**


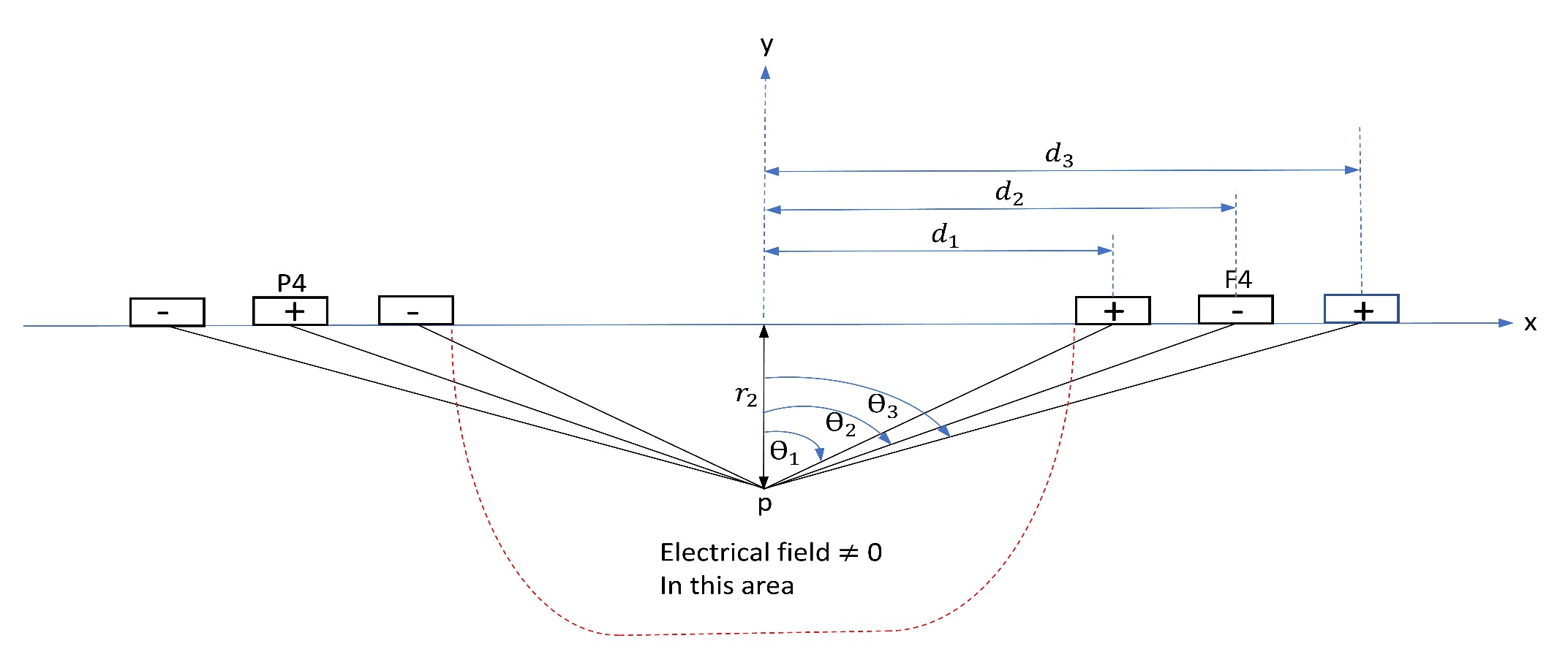


**C**

**Figure S1.** **The electrodes from the sagittal side view.** *d_1_*, *d_2_*, *d_3_*, *d_4_* and *Ɵ_1_*, *Ɵ_2_*, *Ɵ_3_*, *Ɵ_4_* have two points of view; [1] distance and skewed angle from the center electrode to return electrodes in frontal (F4) and parietal (P4) sites which is related to point **A**, [2] distance and skewed angle from the center in between sites to each electrode (F4, P4 and their returns) in frontal (F4) and parietal (P4) sites which is related to point **B**.

**A)** In the in-phase condition, the electric field at point p is dominant from electrodes above its point p; **B)** In the in-phase condition, the electric field at point p position between frontal and parietal is $\approx0$; **C)** The electric field generated by electrodes in anti-phase condition. The electric field at point p position between frontal and parietal is $\neq0$. r_1_ and r_2_ are the distance from the scalp to point p inside the brain.

**References**

Benjamini, & Hochberg. (1995). Controlling the False Discovery Rate: A Practical and Powerful Approach to Multiple Testing. *Journal of the Royal Statistical Society. Series B (Methodological)*.

Labate, G., & Matekovits, L. (2016). Kirchhoff’s current law as local cloaking condition: theory and applications. *Electronics Letters*, *52*(21), 1749–1751. https://doi.org/10.1049/EL.2016.2682

Paul, C. R. (2001). *Fundamentals of electric circuit analysis*. 501.

Saturnino, Madsen, K. H., Siebner, H. R., & Thielscher, A. (2017). How to target inter-regional phase synchronization with dual-site Transcranial Alternating Current Stimulation. *NeuroImage*. https://doi.org/10.1016/j.neuroimage.2017.09.024
